# Hydrated Deep Eutectic Solvents for the Sustainable Valorization of Textile Waste

**DOI:** 10.1002/cssc.71028

**Published:** 2026-09-04

**Authors:** Zoe Smania, Degnet M. Dereje, Silvia Zanatta, Michele Modesti, Pietro Ostellari, Silvia Gross, Mauro Carraro

**Affiliations:** ^1^ Department of Chemical Sciences University of Padova Padova Italy; ^2^ Department of Industrial Engineering University of Padova Padova Italy; ^3^ INSTM UdR University of Padova Padova Italy; ^4^ CNR Institute on Membrane Technology “Enrico Drioli”, Unit of Padova Padova Italy

**Keywords:** cellulose, cotton solubilization, green solvents, hydrated deep eutectic solvents, metal salts hydrates, textile waste

## Abstract

A series of new deep eutectic solvents (DESs) was prepared to enable a greener approach to cellulose recovery from cotton products and textile waste. In this context, while choline‐based binary DESs have been extensively investigated, alternative systems, especially metal‐based hydrated DESs, have received less attention. In this work, four novel ternary DESs were synthesized using (i) ZnCl_2_ or CaCl_2_ and (ii) lactic acid or tartaric acid, (iii) with water added as a third component. By combining the hydrogen bond‐disrupting capacity of traditional binary DESs with the lubricating‐modifier and stabilizing effects of water, low‐viscosity DESs, stable at room temperature, were obtained. These systems demonstrated remarkable versatility in processing cellulose from different sources, enabling complete dissolution of microcrystalline cellulose (MCC) and extensive structural disruption of cotton fibers to recover regenerated cellulose or nanocellulose (NC). The DES formulations were characterized through thermal and rheological analyses, including differential scanning calorimetry (DSC). The thermal stability, morphology, and structural changes of the processed cellulosic materials were investigated using thermogravimetric analysis (TGA), Fourier‐transform infrared (FTIR) spectroscopy, X‐ray diffraction (XRD), polarized light microscopy (PLM), and electron microscopies (TEM and SEM/EDX). Overall, these results highlight the potential of earth‐abundant, cost‐effective metal‐based DESs for the sustainable valorization of cotton‐derived waste.

## Graphical abstract

Hydrated metal‐based deep eutectic solvents (DESs) composed of zinc or calcium chlorides, organic acids, and water were developed as low‐viscosity, room‐temperature‐stable media for the sustainable valorization of cotton waste. These formulations effectively disrupted the cellulose fiber structure and processed microcrystalline cellulose, medical‐grade cotton, and denim, enabling the recovery of cellulose either as regenerated fibers or as nanocellulose.

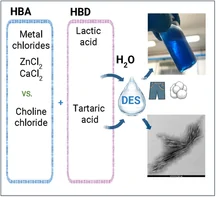

## 1 Introduction

The textile industry ranks among the most environmentally impactful industrial sectors worldwide, owing to its intensive consumption of raw materials, water, and energy. Moreover, its environmental burden is further aggravated by thelow recycling rates and the steady growth in textile waste, a trend exacerbated by rising consumer demand and the rapid turnover inherent in fast‐fashion business models [1]. In Europe, according to EURATEX, households in the European Union spent €282 billion on clothing in 2022, representing a 15% increase compared with the previous year [2]. This increase in textile consumption has been accompanied by a substantial rise in textile waste. In 2025, Europe generated 13.3 million tonnes of postconsumer textile waste, approximately 85% of which was not collected separately but instead entered mixed municipal solid waste streams, where it was ultimately landfilled or incinerated [2, 3].

Postconsumer textile waste is primarily composed of synthetic polymers, above all polyethylene terephthalate (PET), and natural fibers, including cotton, flax, hemp, and jute [4]. Cotton, in particular, accounts for approximately one‐quarter of global textile fiber production [5]. However, its cultivation is resource‐intensive, requiring substantial inputs of water, land, and agrochemicals, while textile manufacturing contributes significantly to environmental pollution [3, 6, 7]. Although cotton is inherently biodegradable under suitable environmental conditions, its end‐of‐life management remains difficult. Recovery and recycling rates are still low, and most postconsumer cotton textiles are disposed of in landfills, where oxygen‐limited conditions and the widespread presence of fiber blends hinder efficient biodegradation. Consequently, there is an urgent need to develop and implement efficient recycling strategies to improve the management and valorization of cellulose‐based textile waste. To address this challenge, the European Commission has recognized textile recycling as a priority through the EU Strategy for Sustainable and Circular Textiles, which aims to achieve textile‐to‐textile recycling and the development of secondary raw material markets by 2030 [8].

Despite advances in textile collection and waste management, cellulose recycling remains challenging. Mechanical recycling, the predominant approach, progressively shortens fibers and degrades their properties, reducing the quality of the recycled material. Chemical recycling has emerged as a promising alternative because it enables the recovery of high‐purity cellulose with preserved functionality. However, its implementation is hindered by the complex supramolecular structure of cellulose, whose extensive intra‐ and intermolecular hydrogen‐bonding network and hierarchical organization confer exceptional mechanical properties, while rendering it insoluble in water and most conventional solvents [9].

In line with the growing demand for more sustainable processing technologies, considerable research efforts have focused on the development of alternative cellulose solvent systems capable of replacing conventional approaches, including cold alkali solutions and N‐methylmorpholine N‐oxide (NMMO), the solvent used in the Lyocell process. In addition, quaternary ammonium‐ and phosphonium‐based solvent systems have been investigated; depending on their chemical structure and counterions, these compounds can form ionic liquids (ILs) capable of dissolving cellulose [10, 11]. Although these solvents have demonstrated significant potential for cellulose dissolution and regeneration, their high cost, technical complexity, and/or demanding synthesis have so far limited their large‐scale industrial implementation.

Recently, several studies have focused on the pretreatment, modification, and dissolution of cellulose using deep eutectic solvents (DESs) [12, 13, 14, 15]. The most widely studied and applied DESs are typically formed by combining hydrogen bond donors (HBDs) and hydrogen bond acceptors (HBAs) [16]. However, a broader range of formulations exists, and they are commonly classified into five main categories (types I–V) according to the nature of their constituent components [17]:

• Type I: a quaternary ammonium salt combined with an anhydrous metal chloride (e.g., ZnCl_2_).
• Type II: a quaternary ammonium salt combined with a hydrated metal halide.
• Type III: a quaternary ammonium salt acting as a HBA combined with a HBD.
• Type IV: a metal salt combined with a HBD, without the presence of a quaternary ammonium salt.
• Type V: nonionic DESs formed from molecular compounds that act as HBD and HBA.

When strong interactions establish between the components, they cause a substantial depression of the melting point relative to that of the individual components, the resulting mixture becomes liquid and remains stable over a wide temperature range. Under these conditions, it is classified as a eutectic solvent [18]. Ideally, DESs remain liquid at or near room temperature, although some systems exhibit melting points as high as 100 °C [19], which still allows their practical use [20]. DESs possess several advantageous properties, including low cost, low volatility, thermal stability, nonflammability, and generally low toxicity. In many cases, they are also bio‐based (i.e., natural deep eutectic solvents, NADES) and biodegradable, making them a more sustainable option with respect to many traditional solvents [21, 22, 23]. Their ability to establish complex intermolecular interactions, combined with their distinctive physicochemical properties, has stimulated extensive research into DESs as versatile and effective media for processing complex materials such as lignocellulosic biomass.

Tong et al. [24] demonstrated that zinc chloride hydrate‐based DESs exhibit high efficiency in dissolving biopolymers, particularly cellulose, with fewer studies also addressing starch, chitin, and silk [25]. Zinc chloride hydrates themselves had previously been recognized for their ability to solubilize moderate amounts of cellulose [26, 27], prompting further exploration of a new class of DESs formulated with low‐impact metal salts to tune the interactions with lignocellulosic materials. Notably, the use of hydrated metal salts in DESs [16] represents an advantage with respect to the use of conventional systems based on quaternary ammonium salts (e.g., choline chloride). In addition to the generally lower cost and wider availability of hydrated metal salts, these systems often exhibit faster cellulose dissolution rates while avoiding several drawbacks associated with conventional DESs, including high viscosity, hygroscopicity, and poor reproducibility [28, 29].

Besides fiber solubilization and regeneration, another opportunity for the valorization of cellulosic fibers consists in their conversion into nanocellulose (NC) [30, 31, 32, 33, 34]. Chemically produced NC is commonly obtained via acid hydrolysis, employing mineral and organic acids such as hydrochloric, phosphoric, nitric, oxalic, and maleic acids, as well as heteropoly acids [35, 36, 37]. More recently, ionic liquids and DESs have also been reported as effective media for the delignification and hydrolysis of cellulosic fibers to produce NC, either as standalone treatments or in combination with subsequent chemomechanical processing steps [32, 33, 38]. Within this context, we previously reported both binary and ternary DESs based on choline chloride and gallic acid—with the optional addition of tartaric acid—for the generation of nanocellulose from cotton fabrics and denim. Yields of up to 85% were achieved after heating at 90 °C for 1 h, followed by 1 h of sonication [39].

In this study, a series of DESs was examined to compare the properties and performance of hydrated metal‐based type IV systems with those of choline chloride‐based type III systems. An initial comparative characterization of their thermal and rheological behavior was conducted to elucidate the distinctive features and potential advantages of the hydrated formulations. The DES compositions investigated in this work originate from a technology disclosed in our filed patent [40], which is here evaluated for cellulose recovery and textile waste valorization. Accordingly, natural carboxylic acids were selected as hydrogen‐bond donors (HBDs) and combined with either choline chloride or calcium or zinc chlorides to enhance overall sustainability and promote the use of abundant, cost‐effective, bio‐based precursors. In some formulations, water was added to reduce the inherent high viscosity of DESs. The selected DESs were subsequently employed to treat cotton‐based materials. Model substrates, including microcrystalline cellulose (MCC), medical‐grade cotton fibers (CF), and two different kinds of blended postconsumer textiles, were used to evaluate cellulose dissolution (MCC) or structural disruption (CF), together with cellulose recovery efficiency. The recovered materials were then comprehensively characterized in terms of structural and morphological properties to identify the most effective formulations and processing conditions for the recovery of cellulose or production of nanocellulose.

Overall, this work introduces several advances in the development of DESs for textile waste valorization. First, previously unreported hydrated type‐IV DESs based on ZnCl_2_ or CaCl_2_ combined with lactic or tartaric acid and water were developed, yielding low‐viscosity solvents that remain liquid at room temperature. Second, these metal‐based DESs were systematically compared with conventional choline chloride‐based DESs under identical experimental conditions. Third, the study demonstrates that, by tuning the DES composition and processing conditions, the same solvent platform can be used (i) to dissolve MCC, (ii) to enable extensive structural disruption of cellulose fibers, or (iii) to produce cellulose nanocrystal (CNC)‐enriched fractions, thus highlighting its versatility. Finally, the methodology was validated using real postconsumer textile waste, i.e., blue denim, extending its applicability beyond model cellulose substrates. To the best of the authors’ knowledge, this is the first report describing hydrated type IV DESs with this dual functionality applied to postconsumer textile waste.

## 2 Experimental Section

### 2.1 Materials

Anhydrous zinc chloride, anhydrous calcium chloride (≥95.0%), L‐(+)‐lactic acid, citric acid, L‐(+)‐tartaric acid, MCC, urea, choline chloride, and ethanol were purchased from Merck and used without further purification. Medical‐grade CF and two different kinds of postconsumer denim (i) 99% cotton and 1% elastan (labeled D01) and (ii) 61% cotton, 36% polyester, and 1% elastan (labeled D02) were considered as cotton sources. D01 and CF were dried at 85 °C for 24 h before use.

### 2.2 DES Preparation

Following established protocols [41], choline chloride was mixed with various HBDs and heated at 80 °C under stirring for 1 h to obtain type III DESs. Type IV DESs were also prepared following a similar procedure, by combining either lactic acid or tartaric acid with selected metal chlorides and water (all new formulations screened, including those not yielding a DES, are reported in Table S1).

Table 1 lists the compositions, molar ratios, and abbreviations of all prepared DESs. Choline‐based DESs were dried under vacuum for 24 h and stored in sealed vials, while salt‐based DESs were used without further purification. All DESs were stored at room temperature. A preliminary comparison of the different DES formulations was done via differential scanning calorimetry (DSC), to study the temperature‐dependent behavior of the DESs, and rheological analysis, to monitor their apparent viscosity and flow behavior. Each DES was then tested for its ability to interact with cellulosic materials [39].

**TABLE 1:** Compositions, molar ratios, and abbreviations of all prepared DESs. The mixtures were heated at 80 °C for 60 min in an oil bath under continuous stirring until homogeneous liquids were obtained.

Label	Components		Molar ratio
Type III DES	HBA	HBD	(HBA:HBD)
CCCA(M)	Choline chloride	Citric acid monohydrate	1:1
CCTA	Choline chloride	Tartaric acid	2:1
CCLA	Choline chloride	Lactic acid	1:1; 1:2; 1:3
CCUr	Choline chloride	Urea	2:1

Type IV DES	Metal salt	Organic acid	(Salt:acid:water)
CaLA	CaCl_2_·H_2_O	Lactic acid	1:1:6
CaTA	CaCl_2_·H_2_O	Tartaric acid	1:1:6
ZnLA	ZnCl_2_·H_2_O	Lactic acid	2:1:4
ZnTA	ZnCl_2_·H_2_O	Tartaric acid	1:0.2:6

### 2.3 Treatment of Cotton‐Based Materials With DESs to Recover the Cellulose

A preliminary solubility assessment was performed by adding increasing amounts of MCC to the prepared DESs. The apparent solubility limit was defined as the maximum cellulose loading that yielded a visually homogeneous solution at 80 °C. Cellulose dissolution was then confirmed by polarized light microscopy (PLM, Figure S5a).

Since ZnLA exhibited the highest cellulose dissolution capability, it was selected for subsequent experiments using different cotton feedstocks, namely, medical‐grade cotton (CF) and denim (D01). The DES, composed of zinc chloride, lactic acid, and water in a 2:1:4 molar ratio, was mixed with the cotton samples (up to 10 wt%) and heated at 50 °C for up to 45 min under continuous stirring in a silicone oil bath. The evolution of fiber disruption during DES treatment was monitored by optical microscopy after 0, 20, and 45 min, while the final extent of cellulose fiber disruption was assessed by PLM (Figure S5b,c). After treatment, 10 mL of deionized water or ethanol was added as antisolvents to dilute the DES and precipitate the cellulose. The insoluble material was recovered by centrifugation (10,000 rpm, 5 min), washed 3 times with deionized water, and vacuum‐dried for 24 h. The recovered cellulose samples were designated ZnLA_CF and ZnLA_D01, corresponding to medical‐grade cotton and denim (D01), respectively.

The blended denim fabric D02, consisting of 61% cotton, 36% polyester, and 1% elastane, was shredded, and its cellulosic (CF_D02, warp) and synthetic (SF_D02, weft) fractions were manually separated. The synthetic fraction was used as a model polyester substrate and treated with ZnLA at 80 °C for 60 min to evaluate its stability in the presence of the DES. Both the treated fibers and the recovered DES were characterized by Fourier transformation infrared (FTIR) spectroscopy before and after treatment.

### 2.4 Conversion of the Cotton Fraction Into Nanocellulose

CF or D01 were immersed (3 wt%) in CaTA, CaLA, and ZnTA to ensure optimal mixing. The mixtures were heated to 100 °C for 60 min. After cooling, the samples were diluted with deionized water and subjected to ultrasonication (Sartorious Stedim Labsonic P homogenizer, with a 3 mm titanium tip and maximum acoustic power 400 W/cm^2^, for a 5 min run at 95% intensity, i.e., 380 W/cm^2^) to obtain a dispersion. The solid was recovered by centrifugation and purified through three successive washing–centrifugation cycles with deionized water (10,000 rpm, 5 min per cycle). The recovered product was then vacuum‐dried for 24 h to yield crystalline nanocellulose (CNC).

### 2.5 DES Recycling

After the initial centrifugation step, during which cellulose or nanocellulose was recovered as the solid fraction, the supernatant containing the DES and the water or ethanol used as antisolvent was collected. The washing solutions generated during the subsequent purification steps were discarded and were not reused. The collected supernatant was concentrated by rotary evaporation (DLAB RE100‐Pro) under reduced pressure at 50–55 °C and 100 rpm to remove the excess water or ethanol. Typically, approximately 48 mL of diluted DES solution (ca. 8 mL DES and 40 mL water) was concentrated within 30 min. Under these conditions, the less volatile DES components (ZnCl_2_ or CaCl_2_ and the organic acid) remained in the liquid phase. The regenerated DES was then weighed to determine the solvent recovery, reconstituted to its original composition by adding deionized water to restore the initial mass of the freshly prepared DES, and directly reused in the subsequent extraction cycle following the same experimental procedure. The performance of the regenerated solvent was assessed by monitoring the cellulose or nanocellulose recovery yield obtained in each recycling cycle with that achieved using the fresh DES.

### 2.6 Characterizations

FTIR spectroscopy was used to characterize DESs and recovered cellulose using either a Nicolet FTIR Nexus spectrophotometer (Nexus Analytics) or a Cary 630 FTIR spectrophotometer (Agilent) in the range of 4000−400 cm^−1^. Spectra were recorded via attenuated total reflectance in absorbance with 4 cm^−1^ resolution and 32 or 64 scans per spectrum.

The melting points of the hydrated metal‐based DESs were measured by DSC on a Q200MFC DSC (TA Instruments) coupled with a Liquid Nitrogen Cooling System on 5–15 mg DESs samples packed in hermetic aluminum pans in a heating–cooling–heating run from –80 °C to up to 60 °C at 10°/min with a N_2_ purge of 50 mL/min. Thermogravimetric analysis (TGA) and relative derivatives (DTG) were performed using a Q5000IR TGA (TA instruments) equipped with an infrared furnace employing four IR lamps and a silicon carbide IR‐absorbent enclosure. Samples—up to 15 mg—were used in high‐temperature platinum‐HT pan. The samples were heated from 40 °C up to 600°C at a heating rate of 10 °C/min, with N_2_ purge of 20 mL/min. Ash content was measured directly after N_2_ purge at 600 °C. Shear viscosities of DESs were measured using a Kinexus Lab+ rotational rheometer equipped with rSpace software and a Peltier plate cartridge for temperature control. A PU20 geometry (20 mm smooth plate) with a fixed 0.3 mm gap was used for all analyses. Two rheological measurement protocols were applied. In time‐dependent experiments, a constant shear rate of 10 s^−1^ was imposed and the apparent viscosity was monitored over time. In shear‐rate‐dependent experiments, the apparent viscosity was measured over a shear rate range of 0.5–500 s^−1^, and the measurements were repeated in triplicate. For comparative purposes, the apparent viscosity values reported at a shear rate of 1 s^−1^ correspond to the second measurement. Additionally, the shear viscosity of ZnLA solutions containing increasing cotton contents was evaluated. All rheological tests were carried out at 25 °C unless specifically stated. The crystallinity of the cellulose samples was determined—in duplicate—via XRD using a Bruker AXS D8 Advance Plus diffractometer equipped with a LYNEXEYE detector in 1D mode. A Cu Kα_1,2_ radiation source (*λ*_1_ = 1.54060 Å, *λ*_2_ = 1.54439 Å, with relative intensity Kα_2_/Kα_1_ = 0.5) was used. X‐rays were generated from a Cu anode powered with 40 kV and a current of 40 mA. Data were collected with Bragg/Brentano geometry in the 10°–65° 2*θ* range, with a scanning rate of 4° min^−1^. The identification of the crystalline phase was performed using the Bruker Diffrac EVA software through a Search and Match procedure. The relative crystallinity index (CrI%) was estimated using the Segal method [42] as follows:

$$\mathrm{CrI}\%=\frac{I_{200}-I_{\mathrm{am}}}{I_{200}}\times 100$$

where *I*_200_ is the peak intensity of the (200) lattice diffraction at 2*θ * ≈ 22.6°, which represents the sum of the crystalline and the amorphous signals, and *I*_am_ is the diffraction intensity of the amorphous fraction at 2*θ *  ≈  18° (as in the maximum intensity reached by the amorphous curve signal). Polarized light microscopy (PLM) observations were performed using a Leica DMI4000B inverted microscope. Transmission electron microscopy (TEM) observation on the CNCs was performed using an FEI Tecnai G2 microscope, operating at 100 kV, equipped with a 4  MP Olympus Veleta camera and a TVIPS F114 camera. A droplet (5 μL) of diluted CNC slurry was dropped on the carbon‐coated electron microscopy grid and negatively stained using 1 wt% phosphotungstic acid solution. Fiber diameters and lengths were evaluated by Image J software, considering at least 100 fibers for sample. The morphology of the samples was characterized by SEM using secondary electron (SE) imaging. Images were acquired at an accelerating voltage of 20 kV, a working distance of 9.9 mm, and a magnification of 101×. Elemental composition was determined by energy‐dispersive X‐ray spectroscopy (EDS) using an X‐Act detector (Oxford Instruments) operated at the same accelerating voltage with a live acquisition time of 48.5 s. The zeta potential was measured using a Malvern Zetasizer Nano‐S instrument equipped with disposable plastic cells. For each measurement, approximately 0.5 mg of the sample was dispersed, upon sonication, in 2 mL of deionized water.

## 3 Results and Discussion

### 3.1 Preliminary DES Evaluation

The DESs were initially characterized to assess and compare their thermal properties and viscosity, as reported in Table 2.

**TABLE 2:** Composition and selected thermal and rheological properties of DESs investigated during the initial screening. Thermal events are reported only when detectable during the first heating cycle by DSC, and apparent viscosity values are given only for systems that remained stable under the applied shear conditions.

Label	HBA	HBD	**Molar ratio (HBA:HBD:H**_**2**_ **O)**	Thermal events		**Viscosities (Pa s) at 25 °C** (Shear rate = 1 s^−1^)
				*T*_g_, °C	*T*_m_, °C	
Type IV DES						
CaLA	CaCl_2_·H_2_O	Lactic acid	1:1:6	n.d.	14.3	n.m.
CaTA	CaCl_2_·H_2_O	Tartaric acid	1:1:6	−14.4		3.880
ZnLA	ZnCl_2_·H_2_O	Lactic acid	2:1:4	−47.1	n.d.	0.227
ZnTA	ZnCl_2_·H_2_O	Tartaric acid	1:0.2:6	n.d.	n.d.	n.m.
Type III DES						
CCCA(M)	Choline chloride	Citric acid·H_2_O	1:1:1	−47.1	n.d.	106.8
CCTA	Choline chloride	Tartaric acid	2:1	−53.2	n.d.	26.43
CCLA	Choline chloride	Lactic acid	1:1	n.d.	52.2	n.m.
CCLA	Choline chloride	Lactic acid	1:2	n.d.	n.d.	n.m.
CCLA	Choline chloride	Lactic acid	1:3	n.d.	n.d.	n.m.
CCUr	Choline chloride	Urea	2:1	n.d.	n.d.	273.8

Abbreviations: n.d., not detected; n.m., not measured.

**FIGURE 1:**
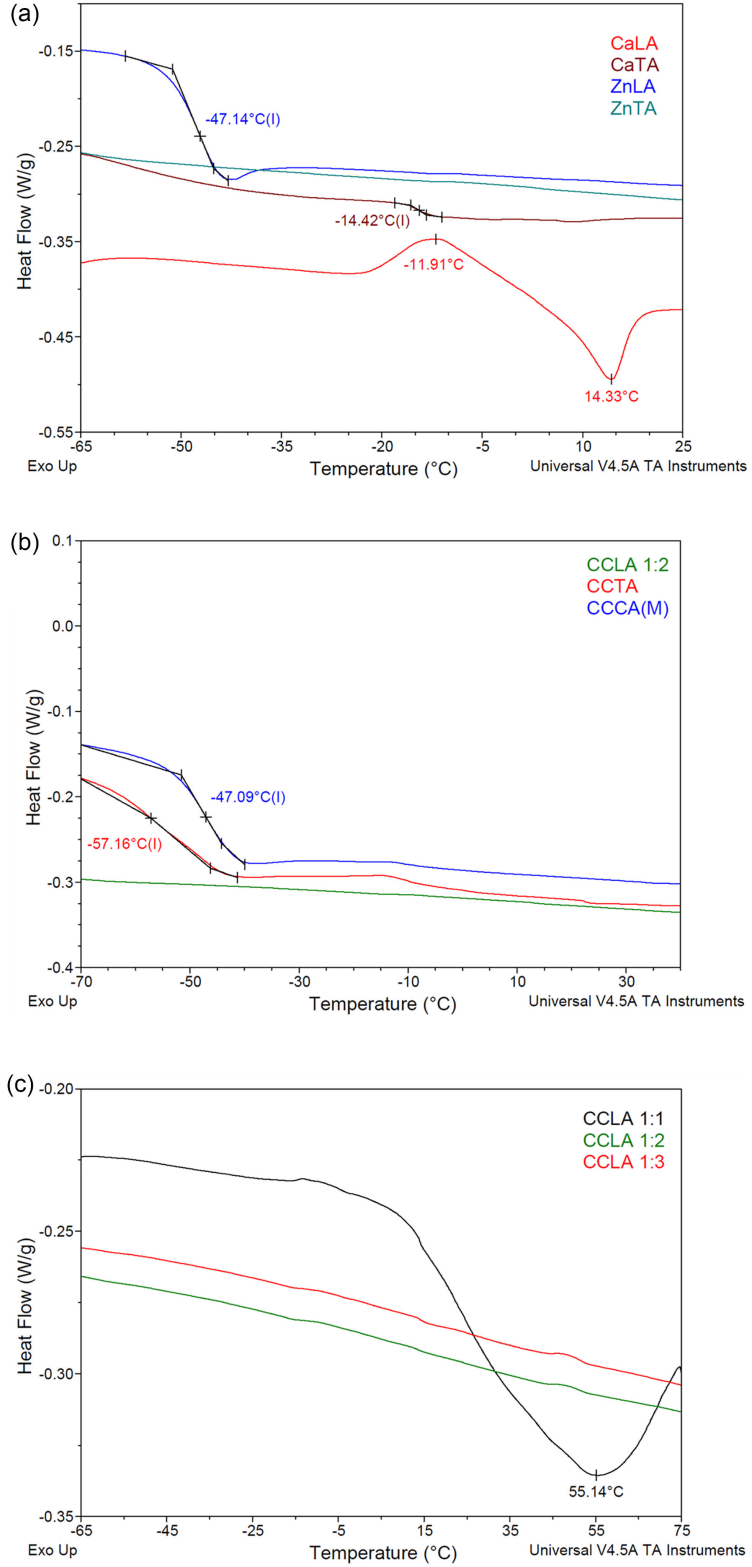
First‐heating DSC thermograms (exo‐up) from a heat–cool–heat program (10°/min) of (a) salt‐based DESs (CaTA, CaLA, ZnTA, ZnLA) and (b) choline‐based DESs (CCTA, CCLA, CCCA(M)) and (c) CCLA prepared at different molar ratios (1:1, 1:2, and 1:3).

All systems were subjected to a preliminary mixing step at 25 °C for 16 h to check whether liquid formation could occur under ambient‐temperature conditions. However, all formulations formed homogeneous liquid mixtures only upon heating to 80 °C. Nevertheless, all mixtures remained homogeneous during storage at room temperature, except for CaLA, which recrystallized after approximately 15 days, and CCLA (1:1), which crystallized immediately upon cooling. In the case of CaLA, the delayed recrystallization may be ascribed to the presence of an extended hydrogen‐bonding network and the associated high viscosity, as reported for similar DES systems [43], which could hinder molecular mobility and slow structural reorganization, especially if the eutectic temperature lies above room temperature. DSC was used to investigate the physical changes of the material in response to temperature by measuring the heat flow absorbed or released by the material against temperature variation. The samples were heated and cooled following a three‐step heating–cooling–heating procedure. Only cycle 1, representing the sample thermal history, is reported (Figure 1a–c and Table 2) since no crystallization was detected during cooling and the second heating fully overlapped the first one. The melting temperature (*T*_m_) of the crystalline fraction is determined as the minimum of the endothermic peak in the heating cycle, while the glass transition temperature (*T*_g_) appears as a step change in the baseline due to a variation in heat capacity. The cold crystallization temperature (*T*_cc_) is identified as the maximum of the exothermic peak observed during heating, corresponding to the crystallization of the previously supercooled or amorphous DES.

Although the hydrated DESs investigated here formally consist of three components (zinc or calcium salt, organic acid, and water), water does not necessarily act as an independent third component in the classical eutectic sense. Instead, it is largely incorporated into the extended hydrogen‐bonding network, behaving as structurally bound water rather than forming a separate aqueous microphase. This interpretation is supported by the DSC thermograms, which show no endothermic transition around 0 °C that would be expected from the melting of free water (ice), indicating the absence of a segregated aqueous phase. The significant influence of water on the phase behavior and thermal transitions of DESs has been well demonstrated in archetypal systems such as choline chloride/urea. In these systems, both the controlled moisture content and the heating/cooling scan rate strongly affect the visibility of phase transitions and the apparent solid–liquid equilibrium [44]. In particular, the melting temperature decreases almost linearly with increasing water content up to approximately 10 wt% relative to the DES.

From a thermodynamic standpoint, a eutectic mixture is defined by a melting temperature lower than that of each pure component. Thus, for binary DESs such as choline chloride–tartaric acid, the eutectic temperature should lie below the melting point of tartaric acid and far below that of choline chloride. This concept also applies to pseudo‐binary systems, in which a hydrated salt effectively acts as the hydrogen‐bond acceptor. However, the absence of a melting endotherm within the investigated temperature range does not necessarily imply that the eutectic temperature lies outside this interval. Indeed, many DESs undergo vitrification upon cooling and fail to crystallize under conventional DSC conditions despite possessing a thermodynamically defined eutectic temperature [45]. The extensive hydrogen‐bonding and electrostatic network restricts molecular mobility, hindering nucleation and growth [46, 47]. Consequently, DSC thermograms typically show a glass transition rather than a melting peak, while the detection of melting transition may require tailored protocols (e.g., annealing or reduced heating rates) [44, 46, 48, 49, 50]. Accordingly, no melting transitions were observed for most salt‐based hydrated DESs (Figure 1a) and choline‐based DESs (Figure 1b,c), except for CaLA and CCLA 1:1. In CaLA (Figure 1a, red curve), an endothermic peak at *T* ≈14 °C appears after cold crystallization (*T*_cc_ ≈ −12 °C). Such behavior is typical of metastable glassy DESs, where crystallization is kinetically hindered upon cooling but becomes possible during reheating above *T*_g_, as sufficient molecular mobility is restored [51]. This thermal response is consistent with the observed tendency of CaLA to crystallize during storage at room temperature, while regarding CCLA (Figure 1c), the 1:1 formulation rapidly solidified at room temperature during preparation, motivating the further investigation of 1:2 and 1:3 molar ratios (CC:LA); consistently, in the corresponding thermograms, the 1:1 mixture of choline chloride and lactic acid shows a broad endothermic event around 50 °C, attributed to the melting of L‐lactic acid [52].

As regards glass transition phenomenon, unlike low‐molecular‐weight compounds that readily crystallize and melt without exhibiting a glass transition, DESs and hydrated DESs form strong cooperative hydrogen‐bonding and ionic networks that reduce molecular mobility and create a frustrated energy landscape. In DSC, *T*_g_ appears as a second‐order transition associated with a step change in heat capacity, marking the onset of cooperative molecular mobility within the amorphous phase [53] and represents the onset of long‐range molecular mobility in these systems. Consistently, numerous studies [45, 46, 49, 50, 54] describe DESs as strong glass formers. This behavior is well documented for choline chloride‐based DESs and systems with strong HBDs, where dense interaction networks hinder nucleation and growth. Among the salt‐based DESs (Figure 1a), CaLA shows no detectable *T*_g_ but undergoes cold crystallization (*T*_cc_) followed by melting (*T*_m_), whereas CaTA exhibits a weak *T*_g_, indicating partial vitrification. For zinc‐based systems, ZnLA presents a well‐defined *T*_g_ at approximately −47 °C, consistent with pronounced glass‐forming behavior, while ZnTA displays no thermal events, suggesting either a *T*_g_ below the experimental limit or a highly rigid network suppressing measurable heat‐capacity changes. A similar variability is observed for choline‐based DESs (Figure ‍1b). CCLA shows no detectable transitions, implying vitrification below −80 °C or kinetic arrest, whereby molecular mobility becomes too limited to produce a resolvable *T*_g_ under the applied experimental conditions. Conversely, CCTA (*T*_g_ ≈ −53 °C) and CCCA(M) (*T*_g_ ≈ −47 °C) exhibit clear glass transitions, confirming their strong glass‐forming character. Overall, these results demonstrate that relatively small compositional changes can markedly influence molecular mobility, dynamic arrest, and the resulting thermal behavior of DESs.

**FIGURE 2:**
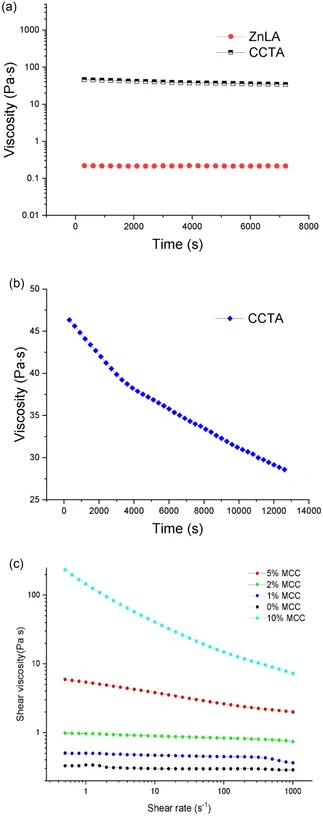
(a) Viscosity of CCTA and ZnLA monitored over time at 25 °C (logarithmic scale) and (b) a detail of CCTA viscosity decreasing as a function of time; and (c) Shear viscosity as a function of shear rate for ZnLA as a function of dissolved MCC content: 0, 1, 2, 5, 10 (wt%) (logarithmic scale).

As both viscosity and thermal behavior can be qualitatively influenced by the extent of molecular mobility within the liquid phase [55], and remarkably affect the practical applicability of these formulations, rheological measurements were performed on a selection of the prepared DESs to characterize their flow behavior (Table 2). CCTA exhibited a 38% decrease in viscosity after 3 h of exposure to air (Figure 2a,b), whereas ZnLA showed a much smaller variation (>2%) under the same conditions. Considering how choline chloride–organic acid DESs are commonly reported to exhibit Newtonian behavior [47, 56], this difference may be attributed to moisture uptake rather than to a modification of the intrinsic rheological profile [57], consistent with the known hygroscopic nature of choline chloride‐based formulations [58]. This moisture sensitivity is often seen as a drawback [59]. In contrast, ZnLA maintained both a stable viscosity and Newtonian behavior throughout the exposure period, indicating a lower sensitivity to ambient humidity. Furthermore, the hydrated metal‐based DESs exhibited substantially lower viscosities than the choline chloride‐based systems. In particular, ZnLA and CaTa showed viscosities of only 0.227 and 3.88 Pa·s, respectively, at 25 °C and a shear rate of 1 s^−1^. These values are one to three orders of magnitude lower than those measured for the choline chloride‐based DESs prepared in this work, whose viscosities ranged from several tens to several hundreds of Pa·s, in agreement with previous reports on quaternary ammonium salt‐based DESs [60, 61].

High viscosity is a well‐documented limitation of many DESs, particularly for systems characterized by restricted molecular mobility, which can negatively affect both processing and handling [62]. The lower viscosities and greater stability observed for hydrated DESs such as ZnLA and CaTA under ambient conditions highlight their considerable potential advantages in applications where fluidity and ease of handling are essential.

### 3.2 Evaluation of MCC Solubility in DES

MCC was initially selected as a model substrate to evaluate the cellulose dissolution capability of the prepared DESs. Different DES compositions and molar ratios were investigated to explore their interactions with cellulosic feedstocks (Table S1). Among the investigated systems, only formulations based on zinc chloride, lactic acid, and water promoted MCC dissolution, with several ZnLA compositions yielding homogeneous cellulose solutions within 24 h at 80 °C.

In particular, the ZnLA formulation with a molar ratio of 4:2.5:9 (ZnCl_2_:lactic acid:water, approximated to 2:1:4 in the following experiments) exhibited the highest apparent MCC solubility alredy after 30 min. In contrast, neither choline chloride‐ nor calcium chloride‐based DESs showed detectable cellulose dissolution under the investigated conditions. Upon addition of a nonsolvent (water or ethanol), the regenerated cellulose formed a transparent gel after standing overnight (Figure S1), consistent with the gelation behavior commonly reported for regenerated MCC [63].

PLM further showed the disappearance of the birefringent MCC particles following DES treatment, indicating disruption of the crystalline cellulose domains and the formation of a homogeneous cellulose‐rich phase (Figure S5a).

The apparent solubility strongly depended on the DES composition, particularly on the acid‐to‐zinc and water‐to‐zinc molar ratios (Figure S2). Molten inorganic salt hydrates are well known to dissolve polysaccharides, with ZnCl_2_·*n*H_2_O representing one of the most effective systems reported [64].

Lu and Shen showed that ZnCl_2_·3H_2_O dissolves up to 5.5 wt% of bacterial cellulose, whereas the *n* = 2 and *n* = 4 hydrates primarily induce swelling rather than dissolution [65]. In addition, Sen et al. reported that at lower hydration levels (*n* = 3), the ZnCl_2_·*n*H_2_O mixture yields [Zn(H_2_O)_6_][ZnCl_4_] [27]. In the ZnLA systems investigated in our study, the highest apparent MCC solubility, up to 12 wt%, was obtained for compositions with a zinc‐to‐water ratio close to ZnCl_2_·2H_2_O and an acid‐to‐zinc ratio of approximately 1:2. Deviations from this composition consistently resulted in lower cellulose solubility.

These observations suggest that MCC dissolution does not originate from a single DES component but rather from the synergistic action of hydrated zinc species and lactic acid. This interpretation is consistent with the DFT study of Tong et al. [24], who showed that interaction energies sufficient to disrupt cellulose hydrogen bonding (16.8–25.5 kcal mol^−1^ [66]) were achieved only when hydrated metal salts, including ZnCl_2_·3H_2_O, were combined with HBDs such as oxalic, acetic, or phosphoric acid, whereas the individual components exhibited much weaker interactions with cellulose.

Within this framework, water plays a dual role. Besides participating in the hydrogen‐bonding network of the DES, it modulates the physicochemical properties of the solvent by reducing viscosity and facilitating mass transport while preserving the supramolecular organization of the hydrated DES. Consequently, cellulose accessibility and diffusion are enhanced without forming a separate aqueous phase. This interpretation is consistent with the DSC results and provides a plausible explanation for the enhanced processability observed upon water addition while maintaining the solvent characteristics essential for efficient cellulose processing.

To further investigate the behavior of ZnLA–MCC mixtures, rheological measurements were performed on formulations containing 0, 1, 2, 5, and 10 wt% MCC (Figure 2c). At cellulose loadings below 5 wt%, the viscosity remained essentially unchanged, whereas higher concentrations produced a progressive increase in viscosity. At the highest MCC loadings, viscosity decreased with increasing shear rate, resembling the shear‐thinning behavior commonly observed in polymer solutions [67, 68, 69]. Such behavior is generally associated with the progressive disruption of physical interactions and chain entanglements under shear [70, 71], although similar rheological responses may also arise from gel‐like dispersions containing residual undissolved cellulose [72].

Overall, the combined apparent solubility, PLM, and rheological data indicate that ZnLA is highly effective in disrupting the crystalline structure of MCC and producing homogeneous cellulose‐rich systems under the investigated conditions, although the presence of small amounts of undissolved cellulose at the highest loadings cannot be completely excluded.

### 3.3 ZnLA‐Induced Structural Disruption of Textile‐Derived Cellulose and CF

The ZnLA formulation with a 2:1:4 molar ratio (zinc chloride:lactic acid:water) was also applied to cotton‐based substrates (CF and D01). ZnLA induced extensive structural disruption of both CF and D01 under significantly milder conditions than those required for MCC, reaching cellulose loadings of up to 10 wt%. The evolution of D01 fiber disruption was monitored by visual inspection (Figure S3), bright‐field microscopy (Figure S4), and PLM (Figure S5).

Bright‐field microscopy revealed the progressive disappearance of the native fibrous organization, leading to well‐dispersed cellulose systems after approximately 45 min at 50 °C (Figure S4). PLM further confirmed the progressive structural changes induced by the DES (Figure S5b,c). Before treatment, both CF and D01 exhibited the characteristic morphology of long, continuous fibers displaying intense birefringence under crossed polarizers, arising from the highly ordered crystalline cellulose microfibrils and their anisotropic arrangement. Following DES treatment, the continuous fiber bundles were replaced by shorter and dispersed cellulose fibers. The effect was more pronounced for D01, where birefringent fragments became less abundant and more uniformly dispersed. In contrast, CF still contained numerous bright birefringent fragments, indicating that a larger fraction of crystalline cellulose domains remained after treatment. Overall, the PLM observations indicate that the DES induced extensive disruption and fragmentation of the native fiber architecture, accompanied by a partial loss of structural order and molecular orientation, rather than complete elimination of crystallinity or complete molecular dissolution.

Within this context, cellulose derived from natural fibers typically exhibits a much higher degree of polymerization (DP > 10,000) than MCC (DP ≈ 70–400) [73, 74], and a higher DP is generally associated with greater resistance to dissolution [75]. At the same time, natural fibers contain a larger fraction of amorphous regions than MCC [31], which are more readily accessible to solvent penetration and structural disruption, as widely reported for cellulose during alkaline and solvent‐based treatments [76]. Thus, two competing effects govern the interaction with the DES. While the high molecular weight and chain entanglement of native cellulose hinder complete molecular dissolution, the amorphous regions facilitate solvent penetration, promoting rapid fiber swelling, disentanglement, and fragmentation. Consequently, ZnLA induced extensive morphological disruption of CF and D01, while residual birefringent cellulose domains remained, indicating that complete molecular dissolution was not achieved under the investigated conditions.

Upon addition of an antisolvent (water or ethanol), the recovered cellulose from D01 and CF formed fibrous, wool‐like suspensions, consistent with the typical morphology of regenerated cellulose [63]. SEM analysis (Figure S6) revealed that the recovered material consisted of dense aggregates of interconnected cellulose fibrils with a rough, irregular surface morphology, confirming the extensive structural reorganization induced by the DES treatment. The recovered D01 retained its characteristic indigo‐blue color, suggesting that the interactions between cellulose and the dye were largely preserved and that no appreciable dye extraction or degradation occurred under the applied conditions.

The milder operating conditions and simpler processing achieved with the ZnLA system highlight its potential for cotton recycling applications. Within this scenario, several laboratory‐scale methodologies for the dissolution and recycling of cotton textiles have recently been reported (Table S2). Ionic liquid‐based processes generally achieve high cellulose recovery from denim but often lead to a substantial reduction in the degree of polymerization (DP) [77]. NMMO‐based systems show the opposite trend, largely retaining DP values but demanding extended pre‐swelling, showing evidence of indigo dye interfering with the dissolution process, and suffering from solvent degradation during processing [78]. Ternary NMMO/DES formulations mitigate some of these drawbacks, although the presence of NMMO remains necessary to improve the dissolution of dyed textiles [79]. A more conventional DES (choline chloride:lactic acid) operates at longer reaction times and higher temperatures than the other processes reported, yielding good cellulose recovery but with substantial DP loss, and without discussing dye behavior [80]. In contrast, the ZnLA system presented here enables fast, mild‐temperature processing with no pretreatment. The presence of indigo did not prevent fiber disruption and cellulose recovery with ZnLA under the investigated conditions. This behavior compares favorably with reports of dye‐related limitations in NMMO‐based processing. Moreover, this combination of low‐temperature operation, dye retention, and potential solvent recovery positions ZnLA as a promising green alternatives for denim recycling.

### 3.4 Characterization of Recovered Cellulose

The recovered (r) materials, after treatment with ZnLA, were labeled rCELL_CF and rCELL_D01 according to the textile feedstock (CF and D01, respectively). FTIR spectra of the samples were compared with those of the corresponding untreated textiles, as shown in Figure S7. The characteristic absorption bands of cellulose at approximately 3300, 2900, 1425, 1375, 1160, 1115, 1060, and 897 cm^−1^ were preserved in both rCELL_CF and rCELL_D01, indicating that the cellulose backbone remained intact following DES treatment [81, 82]. In contrast, a new absorption band appeared at approximately 1733–1735 cm^−1^ in both rCELL_CF and rCELL_D01. The emergence of this band suggests the formation of carbonyl‐containing cellulose species during DES treatment. Comparison with the FTIR spectrum of the fresh DES excludes a significant contribution from free residual solvent, whereas comparison with carboxymethyl cellulose (CMC) reveals a similar carbonyl absorption (Figures S8 and S9). A comparable signal has also been reported for cellulose treated with a CaCl_2_/formic acid/water DES system, where esterification was proposed based on complementary experimental evidence [83]. However, FTIR analysis alone does not provide sufficient evidence to identify the nature or position of the chemical modification. Therefore, further structural investigations, such as solid‐state NMR spectroscopy, will be required to determine whether a similar covalent modification occurs in the present system.

In the denim‐derived sample, the absorption in the 1600–1630 cm^−1^ region was slightly more intense than in the corresponding medical cotton sample (Figure S7). This feature may partly originate from residual indigo dye through aromatic C═C stretching vibrations [84], although overlap with the H─O─H bending vibration of adsorbed water precludes an unambiguous assignment.

**FIGURE 3:**
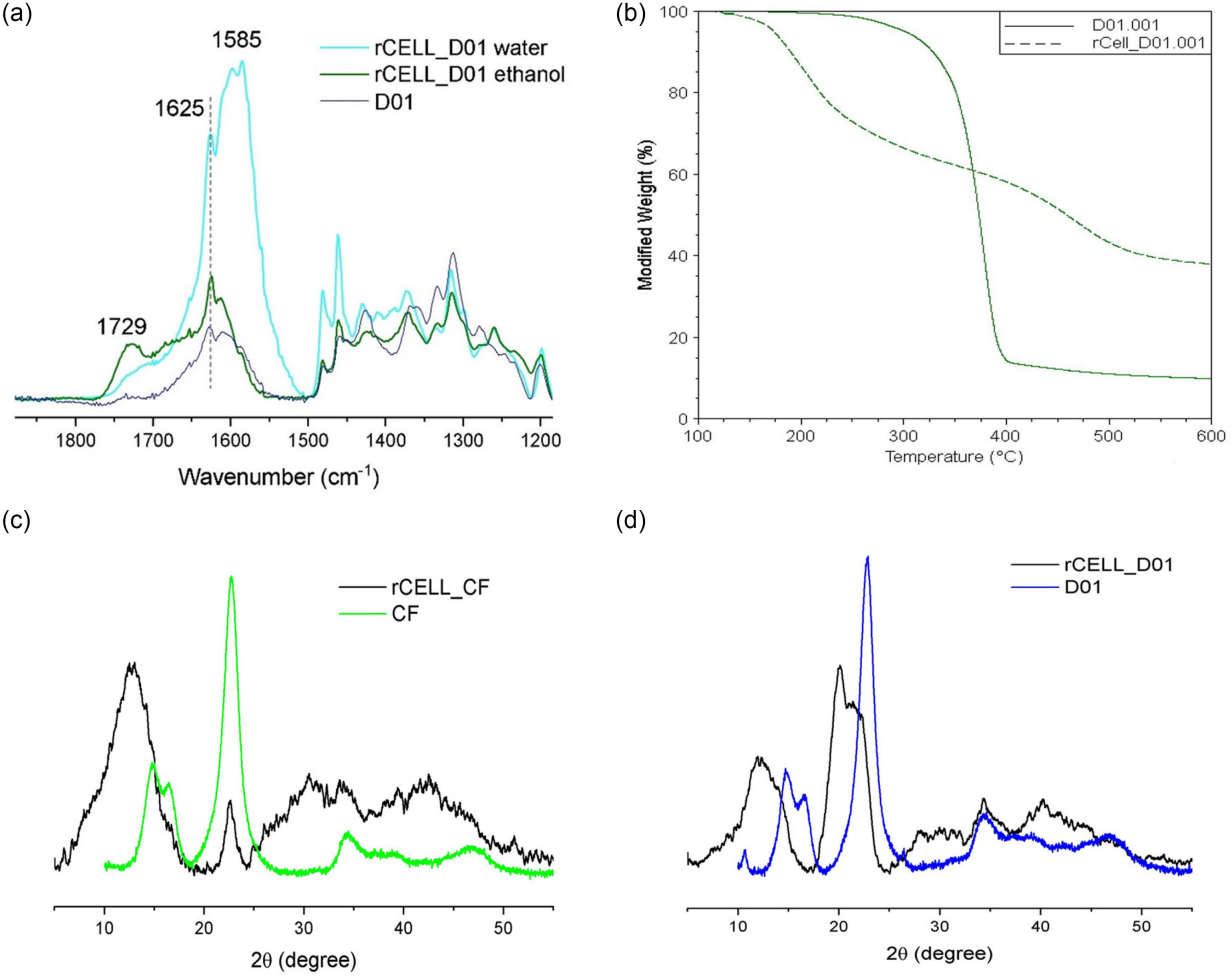
(a) Detail of FTIR spectra of ethanol or water coagulated rCELL_D01, compared with pristine D01, to emphasize the differences in the carbonyl region; (b) thermogravimetric analysis from 100 to 600° of D01 and rCELL_D01 at a heating rate of 10 °C/min in N_2_ atmosphere at 20 mL/min; and (c,d) X‐ray diffractograms for CF and D01, respectively, before and after ZnLA treatment.

As the recovery of cellulose from ZnLA solutions was achieved by the addition of water or ethanol as antisolvents, their influence was investigated by analyzing the FTIR region between 1200 and 1800 cm^−1^ (Figure 3a). Cellulose recovered from D01 using water exhibited a strong band at 1585 cm^−1^, attributable to the asymmetric stretching vibration (*ν*_as_) of deprotonated carboxylate groups (─COO^‐^), likely overlapped with H─O─H bending of adsorbed water (1630–1640 cm^−1^), with the associated *ν*_s_ 1420 cm^−1^, whereas samples recovered using ethanol displayed an intense absorption at 1729 cm^−1^, consistent with carbonyl (C═O) stretching vibrations of ester groups or of protonated carboxylic groups [85]. Beside confirming the occurrence of C═O groups, these differences indicate that the antisolvent and coagulation medium influence the quality of the recovered material, being ethanol a better option to isolate a cellulosic material with fewer ionized functionalities and lower water content.

To assess the crystallinity of the recovered materials, X‐ray diffraction patterns of rCELL_CF and rCELL_D01 were collected on dried samples and shown in Figure 3c,d. Compared with the raw D01 and CF materials, the diffraction patterns of the recovered samples show a clear transformation of the cellulose crystalline polymorph. Specifically, the characteristic ($\bar{11}0$), (110), and (200) reflections of cellulose I, typically observed at 2θ ≈ 14°, 16°, and 22°, are initially present. After regeneration, rCELL_CF showed broader and less intense reflections, together with the disappearance of the sharp cellulose I peak, indicating a marked decrease in crystallinity and a partial transition toward cellulose II [86]. This structural rearrangement is consistent with extensive DES‐induced disruption of the native cellulose structure followed by reorganization during antisolvent precipitation. Indeed, pretreatments that reduce cellulose crystallinity have been shown to enhance dissolution efficiency in various solvent systems [87]. Moreover, cellulose II is the thermodynamically more stable polymorph and is typically formed either by mercerization with concentrated alkaline solutions or by regeneration following cellulose dissolution and antisolvent precipitation [88].

TGA further confirmed the structural differences between the pristine and recovered celluloses. Under a nitrogen atmosphere, both pristine samples (D01 and CF) exhibited the characteristic thermal behavior of cellulose, with a single major degradation event (peak degradation temperatures of 379 and 360 °C, respectively), high thermal stability, and low residual char (<10 wt%) [89]. In contrast, the corresponding recovered celluloses (rCELL_D01, Figure 3b and rCELL_CF, Figure S9a) showed a pronounced reduction in thermal stability. Degradation initiated at significantly lower temperatures (approximately 150 °C for rCELL_D01 and 190 °C for rCELL_CF) and proceeded through broad, multistep degradation profiles. In particular, rCELL_D01 exhibited two degradation events centered at 219 and 460 °C, whereas rCELL_CF displayed a main degradation peak at approximately 223 °C followed by gradual decomposition up to 600 °C. Both recovered celluloses also produced substantially higher char residues, reaching approximately 30 wt% for rCELL_CF. This behavior suggests an increase in the amorphous cellulose fraction, which favors char formation over volatile products, together with the possible presence of residual DES‐derived species, degradation products, or structural modifications introduced during the recovery process.

To complement these analyses, scanning electron microscopy coupled with energy‐dispersive X‐ray spectroscopy (SEM–EDX) was performed to evaluate the presence of residual zinc associated with the recovered cellulose. Zinc was consistently detected in all analyzed regions of rCell_CF (ZnLA DES), with an average content of approximately 0.8 wt% (Figure S6c). These results indicate that residual Zn ions remain associated with the regenerated cellulose even after washing and regeneration. This finding is consistent with previous reports by Zhang et al., who described similar thermal stability behavior for regenerated cellulose obtained from ZnCl_2_‐based dissolution systems [90].

Overall, the combined spectroscopic and thermal analyses confirm that the cellulose backbone is largely preserved after treatment while indicating an increased degree of structural disorder together with subtle changes in the chemical environment of the recovered cellulose. These observations suggest the occurrence of strong interactions between cellulose and the ZnLA DES components, as well as with the possible presence of residual Zn‐containing species following regeneration. Such interactions are reflected in the altered thermal behavior of the recovered cellulose and will require further investigation to elucidate their nature.

### 3.5 ZnLA Compatibility With Noncellulosic Materials

To preliminarily evaluate the behavior of blended materials such as polycotton (i.e., cotton/polyester blends) in the presence of the ZnLA DES, additional experiments were performed using the polyester‐rich fraction (SF_D02), manually isolated in the form of warp, from the blended denim fabric D02. This experiment was designed to assess the chemical stability of the polyester component under the selected treatment conditions rather than to demonstrate selective separation from an intact blended textile. After DES treatment, no visible changes in the fibers were observed, and the polyester fraction was quantitatively recovered. The recovered fibers (rSF_D02) exhibited FTIR spectra identical to those of the untreated material, with the characteristic absorption bands of polyethylene terephthalate (PET) remaining unchanged (Figure S10a). Likewise, the FTIR spectrum of the ZnLA DES showed no appreciable changes after contact with the polyester fibers (Figure S10b). These results indicate that the isolated polyester fraction is largely unaffected by the ZnLA treatment under the investigated conditions. Selective processing of intact cotton/polyester blended textiles remains to be demonstrated and will require further investigation.

### 3.6 Nanocellulose Production

Although only one of the four tested DESs showed significant cellulose solubilization, the remaining solvents provided the opportunity to explore alternative pathways in which partial cellulose hydrolysis may also play a relevant role. Owing to their combined Lewis and Brønsted acidity, salt‐based DESs should be able to hydrolyze the amorphous (noncrystalline) regions of cellulose, thereby releasing crystalline nanostructures.

In particular, CF and D01 fibers (up to 10% wt) were treated with selected type IV DESs at 100 °C for 60 min. The solid fraction, recovered after centrifuging and washing with deionized water, appeared as a fibrous pulp. Subsequent sonication [91] promoted further fragmentation of the treated fibers, leading to the formation of aqueous dispersions exhibiting either a white (in the case of CF) or a bluish (in the case of denim) color. All dispersions remained macroscopically stable for at least 7 days, hinting at adequate colloidal stability for medium‐term storage and subsequent characterization. Table 3 collects and compares the results obtained from the treatment of cellulosic materials aimed at nanocellulose production.

**TABLE 3:** Comparative analysis of products obtained from the treatment of cellulosic materials with salt‐based DES, based on yield, degradation temperatures, char content, and crystallinity index.

Cotton feedstock/name	DES	Yield, wt%	***T***_**peak**_ **, °C**	Char, wt%	CrI, %
CF		—	350	4.6	66
CaTA_CF	Calcium chloride:tartaric acid:water (1:1:6)	83	338	7.3	86
CaLA_CF	Calcium chloride:lactic acid:water (1:1:6)	78	337	15.9	85
ZnTA_CF	Zinc chloride:tartaric acid:water (1:0.2:6)	68	203–467	38.0	74
D01		—	379	9.1	67
CaTA_D01	Calcium chloride:tartaric acid:water (1:1:6)	75	371	10.5	87
CaLA_D01	Calcium chloride:lactic acid:water (1:1:6)	68	372	6.6	86
ZnTA_D01	Zinc chloride:tartaric acid:water (1:0.2:6)	72	266–367	20.6	87
MCC					81

**FIGURE 4:**
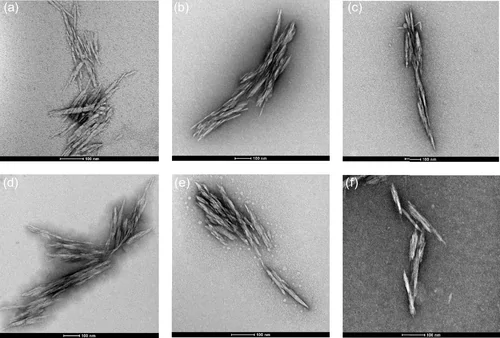
TEM analysis for (a) CaTA_CF, (b) CaTA_D01, (c) CaLA_CF and (d) CaLA_D01, (e) ZnTA_CF, and (f) ZnTA_D01, obtained from aqueos dispersion upon staining with 1 wt% phosphotungstic acid solution. Scale bar: 100 nm.

TEM (Figure 4) was used to evaluate the morphology and the dimensions of the recovered cellulose nanostructures after the treatment. The presence of elongated rod‐like fragments confirms the effectiveness of the DES‐assisted process in producing CNCs from cotton‐based materials, which were labeled according to the solvent system and feedstock employed (CaTA_CF, CaTA_D01, CaLA_CF, CaLA_D01, ZnTA_CF, ZnTA_D01). The particles exhibited lengths ranging from approximately 90–250 nm and widths between 8 and 15 nm and were predominantly organized into bundles rather than appearing as isolated nanocrystals. The highest aspect ratios (15–25) were observed for the CaLA‐derived nanostructures. For comparison, cotton‐derived CNCs are typically reported to have diameters of 1–10 nm, lengths of 100–150 nm, and aspect ratios ranging from 10 to 30 [92].

Within this context, zeta potential measurements were performed on aqueous suspensions of CaLA_CF and ZnTA_CF, both of which exhibited values of approximately −20 mV (Figure S11b,c), indicating moderate colloidal stability. These values are substantially more negative than that measured for rCell_CF prepared using the ZnLA DES (approximately −7 mV, Figure S11a). The higher negative surface charge suggests stronger electrostatic repulsion between the nanocellulose particles, thereby reducing their tendency to aggregate or reassemble. Although many conventional methods rely on the introduction of charged surface groups (e.g., sulfate half‐esters or carboxylate groups) to stabilize CNC suspensions, the moderate colloidal stability observed here was achieved while largely preserving the native chemical structure of the cellulose. Conversely, the lower absolute zeta potential of the ZnLA‐derived sample is expected to facilitate the reassembly of cellulose chains, ultimately favoring the formation of regenerated cellulosic fibers.

All DESs allowed to obtain CNC with yield above 67%. Variations in recovery efficiency are likely attributed to differences in the extent of removal of amorphous cellulose domains and residual noncellulosic components during treatment [91]. In general, D01 samples exhibited slightly lower or comparable yields relative to CF, which may reflect differences in accessibility and purity of the cellulose in the starting material. Among the investigated systems, CaTA afforded the highest CNC yields for both CF and D01 (83% and 75%, respectively), followed by CaLA (78% and 68%, respectively), whereas Zn‐based DESs resulted in comparatively lower recoveries, with ZnTA yielding lower amount of CNC (68% for CF and 72% for D01). These data indicate a higher extraction efficiency for calcium‐based systems under the applied conditions.

**FIGURE 5:**
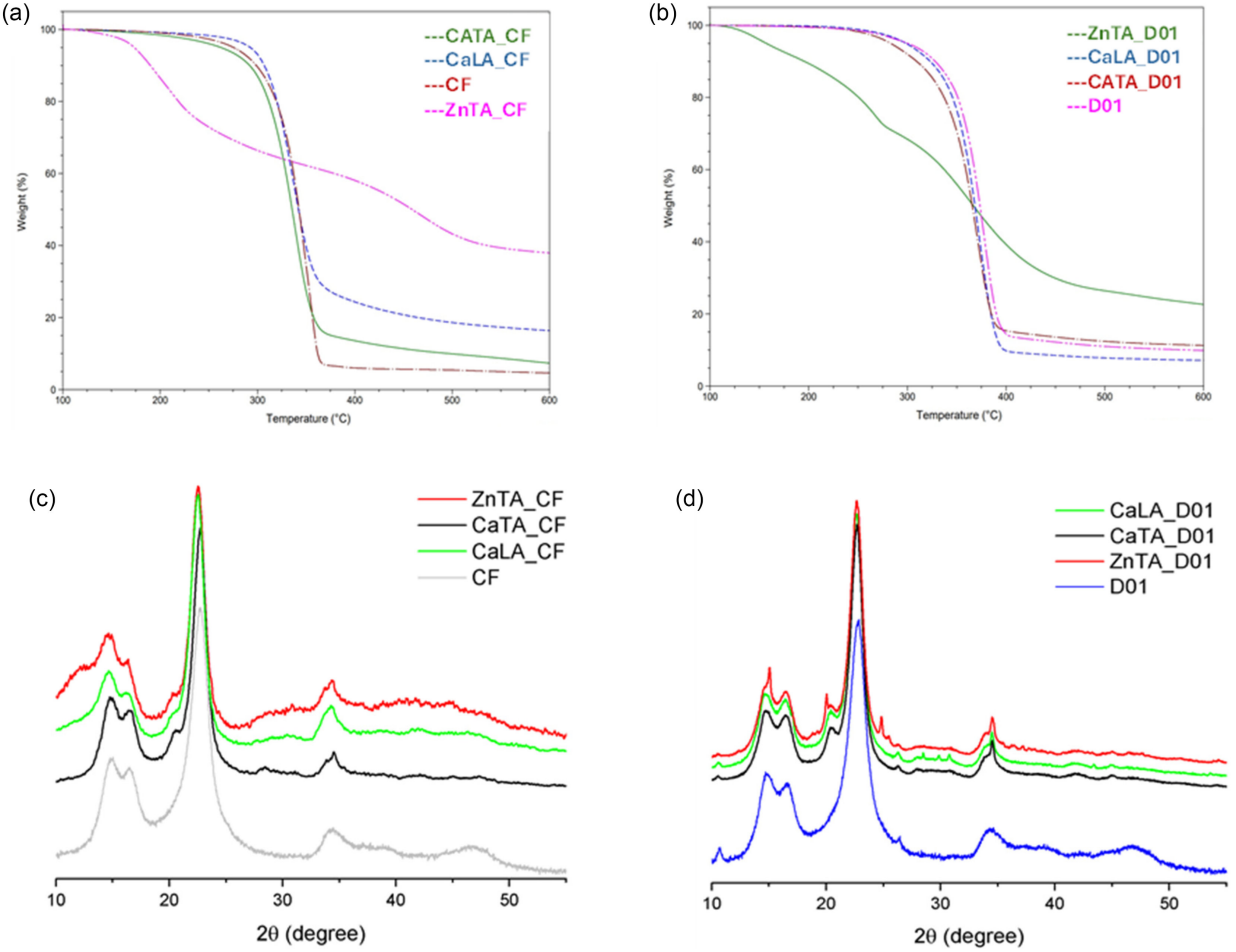
Thermogravimetric analysis from 100 °C to 600° at a heating rate of 10 °C/min in N_2_ atmosphere at 20 mL/min for (a) CF and (b) D01. XRD diffractograms of the obtained CNCs samples in the range 2*θ* = 10°–55° compared with the pristine materials for (c) CF and (d) D01.

Thermogravimetric analyses (Figure 5a,b and Table 3) showed that the CNC samples possessed slightly lower thermal stability than the parent CF and D01 fibers, a trend commonly attributed to their lower degree of polymerization and higher specific surface area. The CNCs obtained using the calcium‐based DESs exhibited similar thermal decomposition behavior, with the main decomposition occurring between 337 and 371 °C, in agreement with values reported for CNCs prepared by hydrolytic methods [92, 93]. These results suggest that the DES treatment did not introduce significant chemical modifications beyond cellulose chain scission, consistent with its intended role in nanocellulose extraction. In contrast, the ZnTA‐derived CNCs exhibited a distinct thermogravimetric profile, characterized by an initial mass loss beginning at approximately 100 °C, followed by a lower thermal stability and a residual char yield exceeding 30 wt% at 600 °C. As in the case of ZnLA, this may be due to thermally labile carbonyl‐containing functionalities (esters and/or carboxylic acids) or residual DES components.

X‐ray powder diffraction was performed to monitor modification in CNC crystalline domains after treatment and to estimate the crystallinity index (CrI%) via the Segal method [42]. As shown in Figure 5c,d, CNC samples displayed characteristic diffraction peaks at 2*θ* = 15.4° ($\bar{11}0$), 16.5° (110), and 22.4° (200), consistent with cellulose I polymorph. In addition, all treated samples exhibited a weak reflection at approximately 20.5°, which has been associated with cellulose II contributions, and it is also present in the reference material (MCC, Figure S12) [25]. ZnTA‐derived samples, particularly ZnTA_D01, exhibited broader diffraction peaks together with an irregular/elevated background intensity, which may reflect a combination of reduced crystallinity and the presence of residual amorphous material, in agreement with the anomalous thermal behavior previously discussed for zinc‐treated nanocellulose.

CrI values (Table 3) increased for all CNCs compared to the corresponding raw fibers. Considering that the CrI of CF and D01 was approximately 25% lower than that of the resulting CNCs, the treatment effectively enriched the obtained material in crystalline fraction. This increase is consistent with the expected removal of amorphous regions—upon hydrolytic reactions—during DES processing, yielding a more crystalline product (for comparison, natural fiber crystallinity typically ranges from 30% to 70%, depending on plant species and growing conditions [94] and can be promising for many advanced material applications. Despite signs of contamination in ZnTA CNCs, crystallinity appeared only moderately reduced in CF‐derived samples (74%), or similar for D01‐derived CNCs, with respect to the other DES.

FTIR analysis indicates that no fundamental structural changes occurred after the treatment besides the partial removal of the amorphous phase. The spectra of the prepared CNCs (Figure S13) showed the characteristic absorption bands of cellulose [81, 82]: at approximately 3300 cm^−1^, the stretching vibration of O─H groups involved in strong H‐bonding in cellulose type I, at 2900 cm^−1^, the symmetric C─H vibrations, at 1425 cm^−1^, the symmetric bending of CH_2_ related to cellulose type I, at 1375 cm^−1^, the bending vibrations of the C─H and C─O groups of the polysaccharide, at 1160 and 1115 for the C─O─C asymmetric vibrations associated with cellulose I and cellulose II polymorphs, respectively. Moreover, the bands at 1060 and 897 cm^−^¹ were assigned to C─O stretching modes within the β‐1,4‐glycosidic linkages and symmetric C─O─C stretching vibrations of β‐glycosidic linkages, respectively. This strong correlation between spectra indicates that the obtained nanocellulose displays functional groups similar to CF and D01, while ZnTA‐derived nanocellulose displayed a weak additional peak at 1736 cm^−1^, in line with what previously observed for ZnLA‐recovered cellulose. CF‐derived samples show a low‐intensity, broad feature at 1630 cm^−1^, consistent with tightly bound moisture. For the D01‐derived samples, the absorption observed in the 1600–1630 cm^−1^ region is more intense, likely due to the additional contribution of residual indigo dye, whose characteristic conjugated carbonyl (C ═O) stretching and aromatic C═C vibrations overlap with the cellulose and water absorption bands [84].

SEM images of the nanocellulosic materials (Figure S14) reveal highly agglomerated fibrillar aggregates, which are consistent with strong intermolecular hydrogen bonding between cellulose surfaces during drying. Although both samples exhibit a similar aggregated morphology, subtle differences are evident. The CaLA_CF sample (Figure S14a,b) forms denser and more compact aggregates with relatively smooth fracture surfaces, whereas ZnLA_CF (Figure S14c,d) displays rougher, less densely packed aggregates with a more open morphology. These observations suggest that the DES composition influences the aggregation and packing of the regenerated nanocellulosic material during drying while maintaining its fibrillar character. EDX spectra collected from different regions of each sample (Figure S15) showed carbon and oxygen as the predominant elements, consistent with the cellulose‐rich nature of the recovered nanocellulosic materials. No clear Ca or Zn signals attributable to residual DES components were detected, suggesting that the washing procedure effectively reduced metal‐containing residues at the analyzed surfaces. However, given the anomalous TGA behavior, the absence of detectable Ca or Zn in EDX should not be interpreted as definitive evidence for complete removal of inorganic residues because EDX is limited by its detection sensitivity and probes only the near‐surface region. Trace Na was instead detected, most likely arising from residual inorganic impurities introduced during sample handling or washing (a weak Si signal was also observed and is attributed to the silicon substrate/sample holder rather than to the CNCs themselves). Overall, the EDX results indicate a predominantly cellulose‐based surface composition and suggest efficient removal of DES‐derived metal ions, although complementary bulk‐sensitive analyses would be required to fully exclude residual inorganic species.

### 3.7 DES Recycling

Solvent recovery is a key factor in the practical implementation of solvent‐based recycling technologies. Therefore, the recyclability of the hydrated metal‐based DESs was preliminary investigated through consecutive extraction cycles (Scheme S1). The ZnLA DES maintained satisfactory extraction performance over two reuse cycles; however, further recycling resulted in a marked decline in extraction efficiency (below 50%), suggesting progressive changes in the solvent system, likely associated with impurity accumulation and variations in solvent composition, leading to partial disruption of the hydrogen‐bonding network. In contrast, the CaLA DES exhibited superior recyclability, maintaining nanocellulose (CNC) yields of approximately 70% over four consecutive extraction cycles (including the fresh‐solvent run, Figure S16), demonstrating that solvent reuse did not significantly compromise cellulose recovery.

After each extraction cycle, the DES was regenerated by removing the excess water added during nanocellulose precipitation via rotary evaporation. The recovered solvent was weighed, and deionized water was added to restore the initial total mass before reuse. Although the recovered DES exhibited a gradual decrease in mass (Figure S16), these losses are most likely attributable to incomplete solvent recovery during handling rather than to a systematic loss upon recycling.

FTIR analysis of the regenerated CaLA DES after the first, second, and third recycling cycles revealed no appreciable spectral changes compared with the fresh solvent (Figure S17). The characteristic absorption bands remained essentially unchanged, indicating that no detectable chemical degradation of the DES components occurred under the applied regeneration conditions. Nevertheless, FTIR alone cannot exclude subtle compositional changes arising from contamination or deviations from the original solvent composition during reconstitution. Therefore, complementary analyses, including viscosity measurements, DSC, and quantitative compositional analysis, will be required to fully assess solvent stability during prolonged recycling.

All in all, these results demonstrate the feasibility of short‐term DES reuse while highlighting the importance of developing more efficient regeneration and purification strategies to preserve solvent performance during extended recycling and support the industrial implementation of DES‐based cotton recycling technologies.

## 4 Conclusions

Our study successfully identified and characterized four brand‐new hydrated DESs, investigating their physicochemical properties and implications for cellulose processing. Thermal analysis revealed different phase behaviors among the systems, with most of the explored DESs acting as glass formers. ZnLA and CaTA, selected for rheological analysis, exhibited comparatively low apparent viscosities, highlighting a potential for industrial applications where fluid handling is pivotal. ZnLA proved particularly effective for processing both MCC and cotton‐based textile fibers, inducing the partial transformation of cellulose I into cellulose II. While PLM indicated extensive solubilization of MCC, medical‐grade cotton and postconsumer denim underwent substantial fiber disruption and fragmentation under mild processing conditions, enabling the recovery of regenerated cellulose.

Post‐treatment analysis showed that the recovered cellulose retained its structural and morphological identity while exhibiting changes in its thermal behavior and FTIR spectra, possibly due to chemical modifications due to interaction with the solvent.

ZnLA also demonstrated that the isolated synthetic fiber‐rich fraction (mainly polyethylene terephthalate) remained largely unaffected under the investigated conditions, supporting further investigation of this DES for the selective processing of intact cellulose‐containing blended textiles.

Furthermore, the combination of three hydrated ternary DESs exhibiting mild hydrolytic properties with ultrasonication proved effective for the production of CNC. Using postconsumer medical‐grade cotton and blue denim as feedstocks, CNC was obtained in high yields (68–83 wt% relative to the starting material) and exhibited higher crystallinity than the parent cellulose fibers. Among the hydrated DESs investigated, the calcium‐based formulations consistently outperformed their zinc‐based counterparts in terms of CNC yield while exhibiting a lower propensity for chemical modification, making them particularly attractive for future process implementation and scale‐up. This superior performance may be attributed to the weaker Lewis acidity and lower coordination strength of Ca^2+^ relative to Zn^2+^. The more labile interaction of Ca^2+^ with cellulose hydroxyl groups appears sufficient to disrupt the intermolecular hydrogen‐bonding network and promote fibril disintegration while minimizing undesired chemical modifications of the polysaccharide. In contrast, the higher Lewis acidity and greater coordination affinity of Zn^2+^ for oxygen‐containing functional groups may contribute to stronger interactions with cellulose during processing. This interpretation is consistent with the more pronounced FTIR spectral changes, the altered thermal behavior observed by TGA, and the presence of residual zinc detected by SEM–EDX.

Although the DESs could be recycled and reused for at least two (Zn‐based) or three (Ca‐based) cycles without a noticeable loss of performance, the present results represent only a preliminary proof‐of‐concept demonstration of solvent reuse rather than a fully validated closed‐loop recovery process. Future work should focus on improving solvent regeneration, long‐term recyclability, process scale‐up, mass balance analysis, and the selective treatment of blended textile waste to further enhance the economic feasibility and environmental sustainability of this recycling strategy.

## Funding

This study was supported by NextGenerationEU through the MICS (Made in Italy – Circular and Sustainable) Extended Partnership (Italian PNRR—M4 C2, Invest 1.3—D.D. 1551.11‐10‐2022, PE00000004) and Dipartimento di Scienze Chimiche, Università degli Studi di Padova (P‐DiSC#03BIRD2025‐UNIPD).

## Conflicts of Interest

The authors declare no conflicts of interest.

## Supporting information

Supplementary Material

## Data Availability

The data that support the findings of this study are available from the corresponding author upon reasonable request.
