# Supplementary material for: Hydrated Deep Eutectic Solvents for the Sustainable Valorization of Textile Waste
Source: ChemSusChem. 2026 Sep 4;19(17):e71028. doi: 10.1002/cssc.71028 (PMC13544636; doi:10.1002/cssc.71028)
Supplement: Supplementary file 1 [file CSSC-19-e71028-s001.pdf]

Supplemental Information for

Hydrated deep eutectic solvents for the sustainable valorisation of textile waste

Zoe Smania[1], Degnet M. Dereje[1], Silvia Zanatta [2], Michele Modesti [2], Pietro Ostellari [1], Silvia Gross [1,3] *, Mauro Carraro [1,4] *

1 Department of Chemical Sciences, University of Padova, Via F. Marzolo 1, 35131 Padova, Italy.

2 Department of Industrial Engineering, University of Padova, Via F. Marzolo, 9, 35131 Padova, Italy.

3 INSTM, UdR University of Padova, Via F. Marzolo 1, 35131 Padova, Italy.

4 CNR Institute on Membrane Technology "Enrico Drioli", Unit of Padova, Via Marzolo, 1, 35131 Padova, Italy.

E-mail: mauro.carraro@unipd.it, silvia.gross@unipd.it

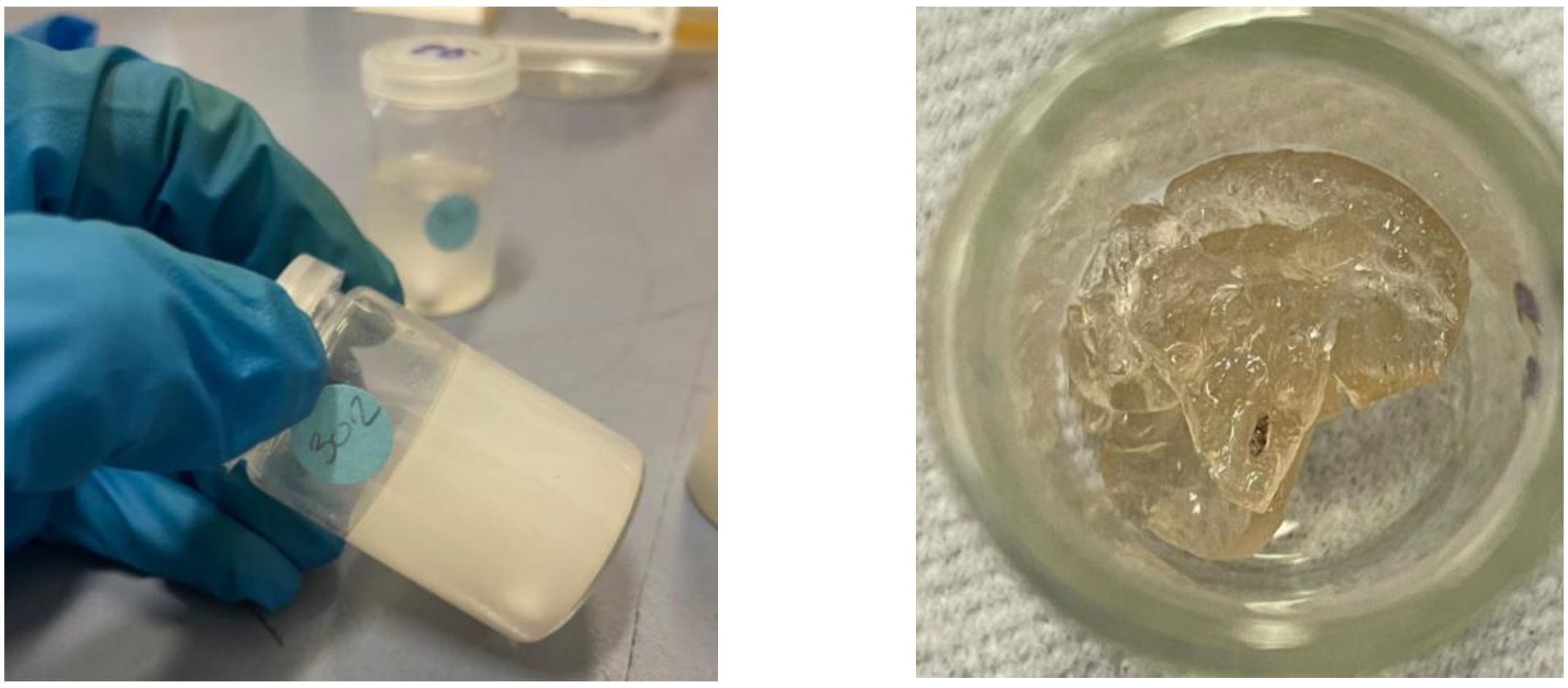

Figure S1: Gel-like behaviour of ZnLA-MCC suspension after addition of water.

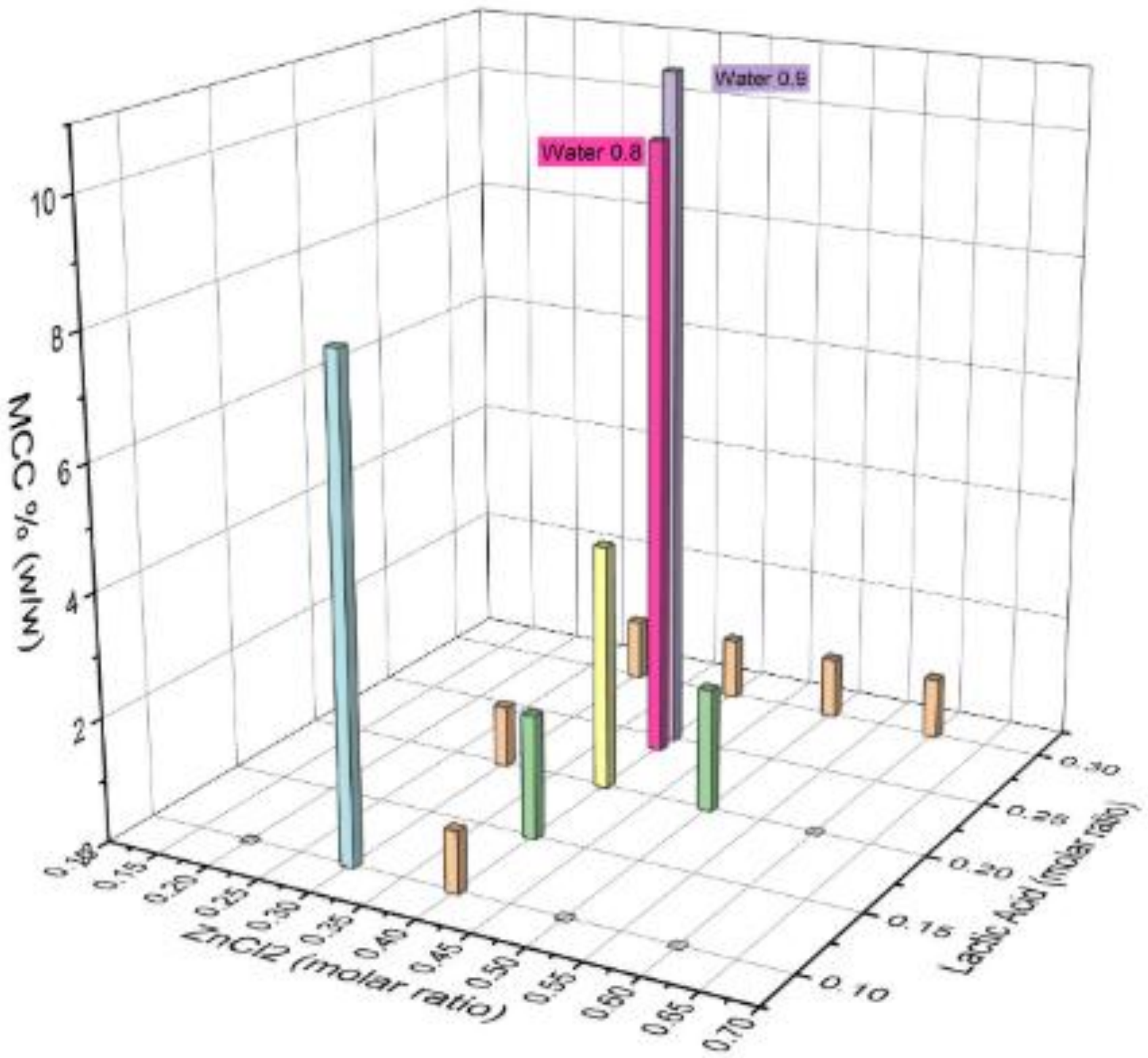

Figure S2: MCC solubility (wt% in DES) trend for different lactic acid (x) and zinc chloride (y) molar ratios within the DES formulation for a fixed water content (i.e. 0.8).

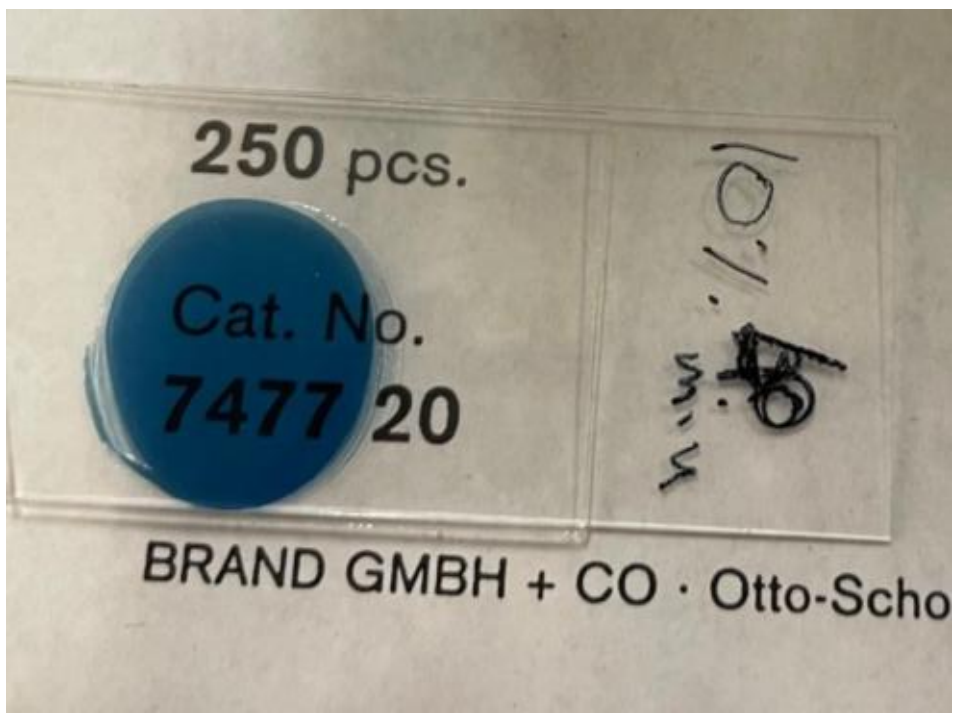

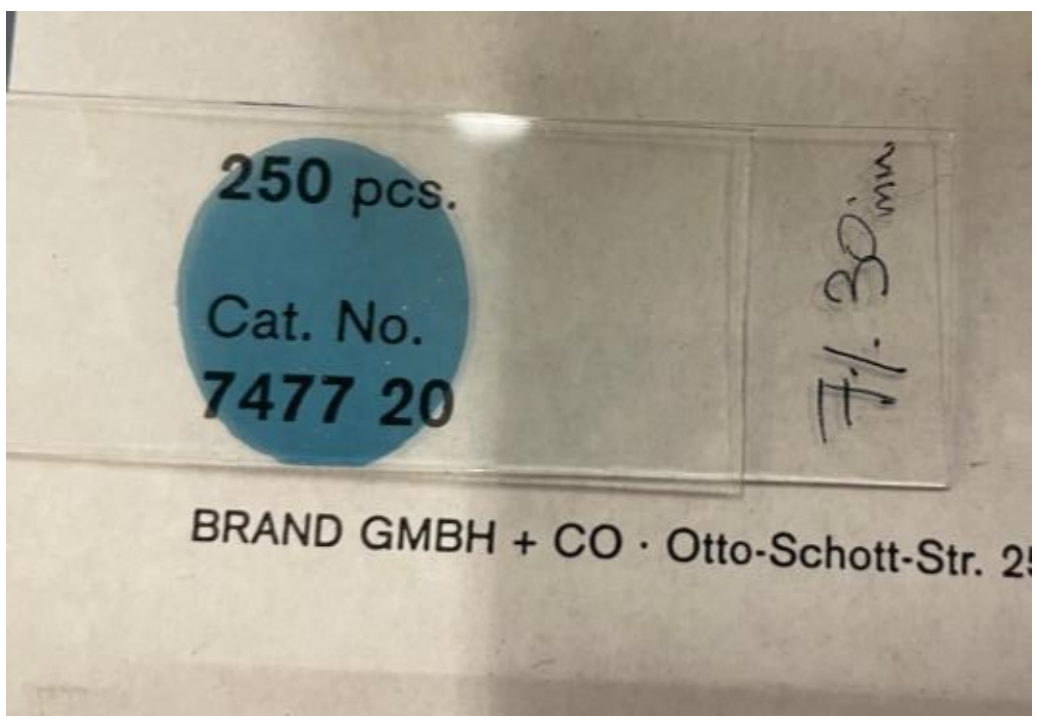

a) b)

Figure S3: Visual assessment of solution transparency. A drop of ZnLA was placed on a glass slide over printed text with a) 10 wt% and b) 7 wt% D01. The high degree of legibility indicates optical transparency of the solution.

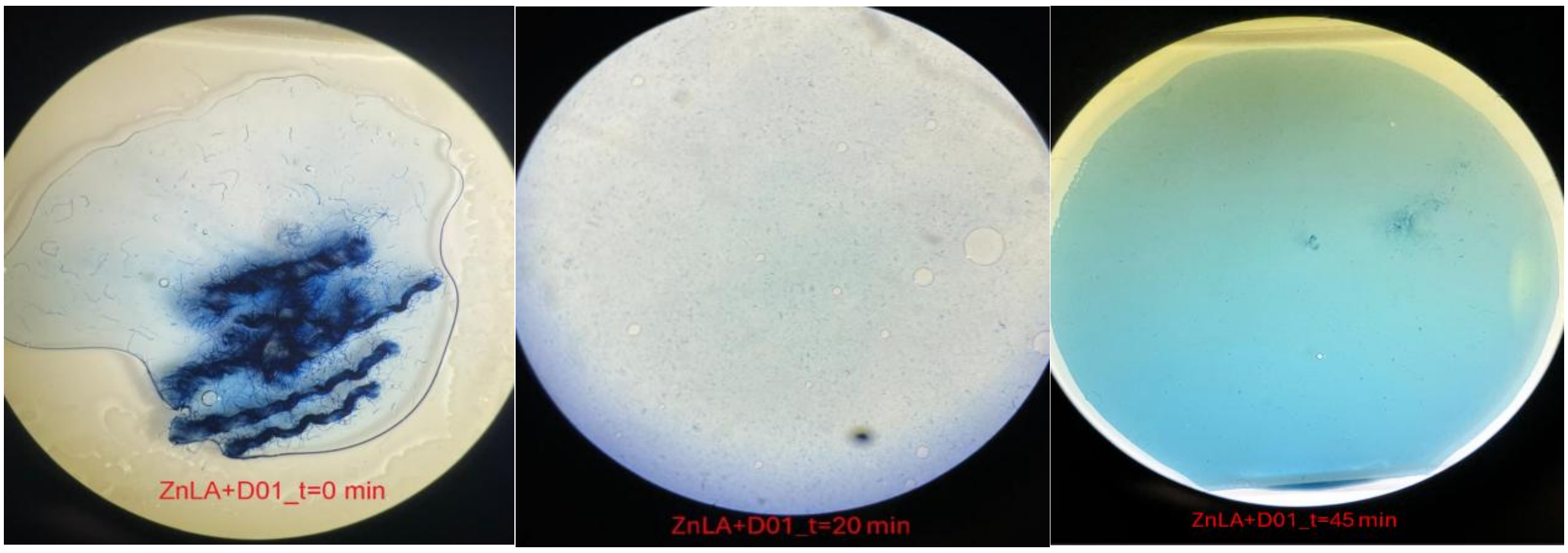

a) b) c)

Figure S4: The dissolution of D01 (and CF) samples was monitored by brightfield optical microscopy. A drop of a ZnLA/D01 solution was placed on a glass slide after 0, 20 and 45 minutes at 50°C. For a 10 wt% cellulose loading of D01, complete disappearance of cellulose aggregates was observed after 45 min at 50 °C, while lower loadings (7 wt%) required approximately 30 min.

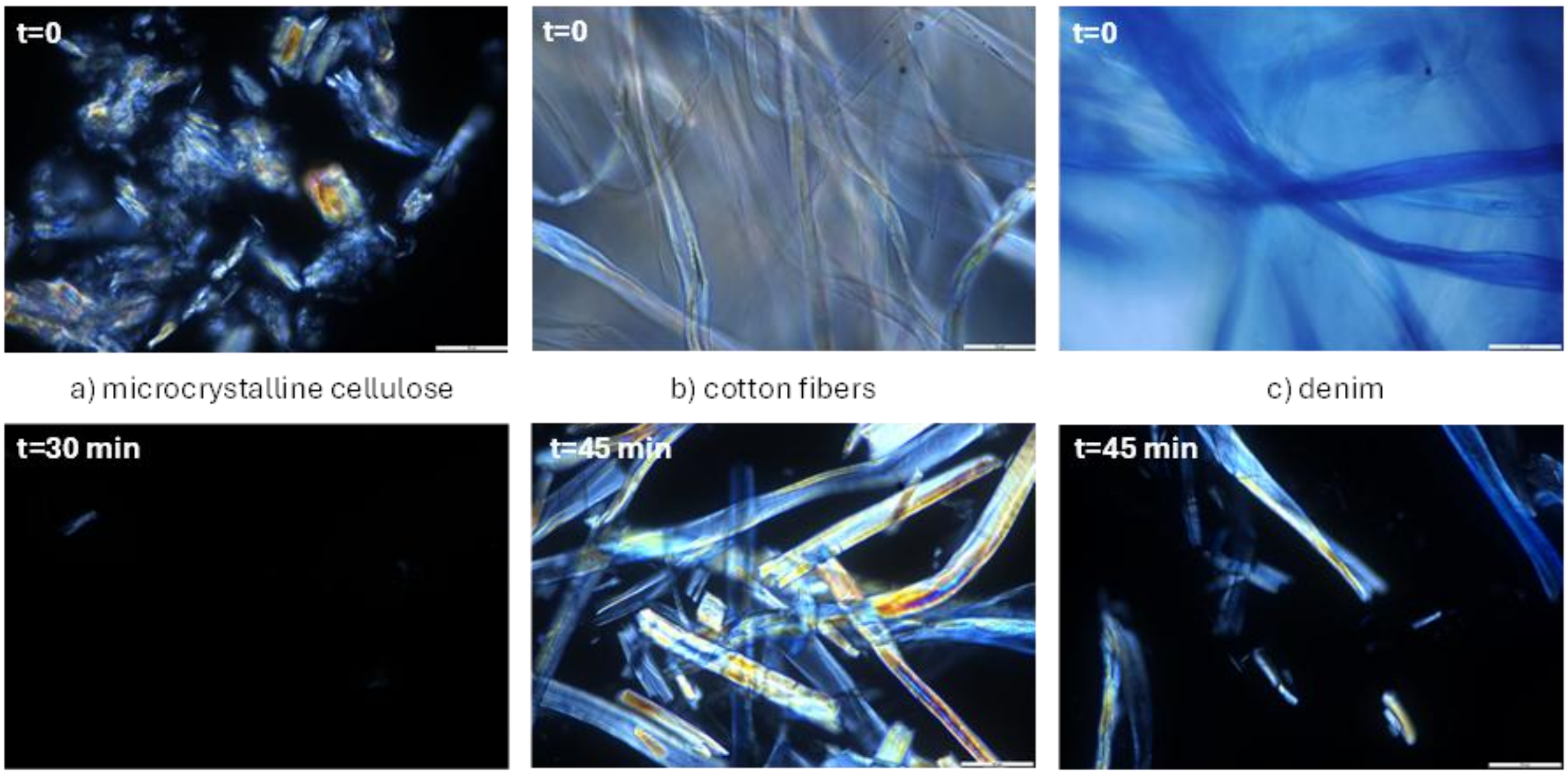

Figure S5: Cross-polarized light microscopy (PLM) images acquired using a Leica DM6 B polarizing microscope showing the dissolution behavior of (a) microcrystalline cellulose (MCC), (b) cellulose fibers (CF), and (c) denim in the ZnLA DES. Top images collected at t=0 min; bottom images collected at t=30/45 min. Scale bar=50 μm.

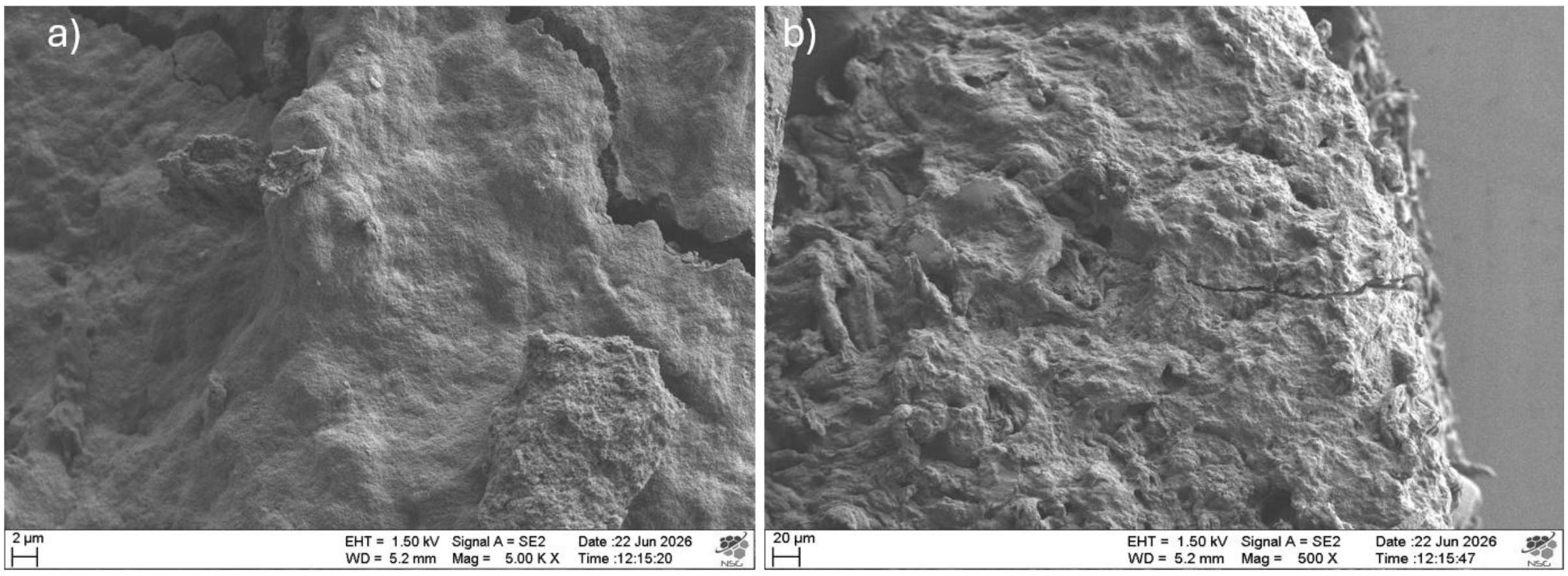

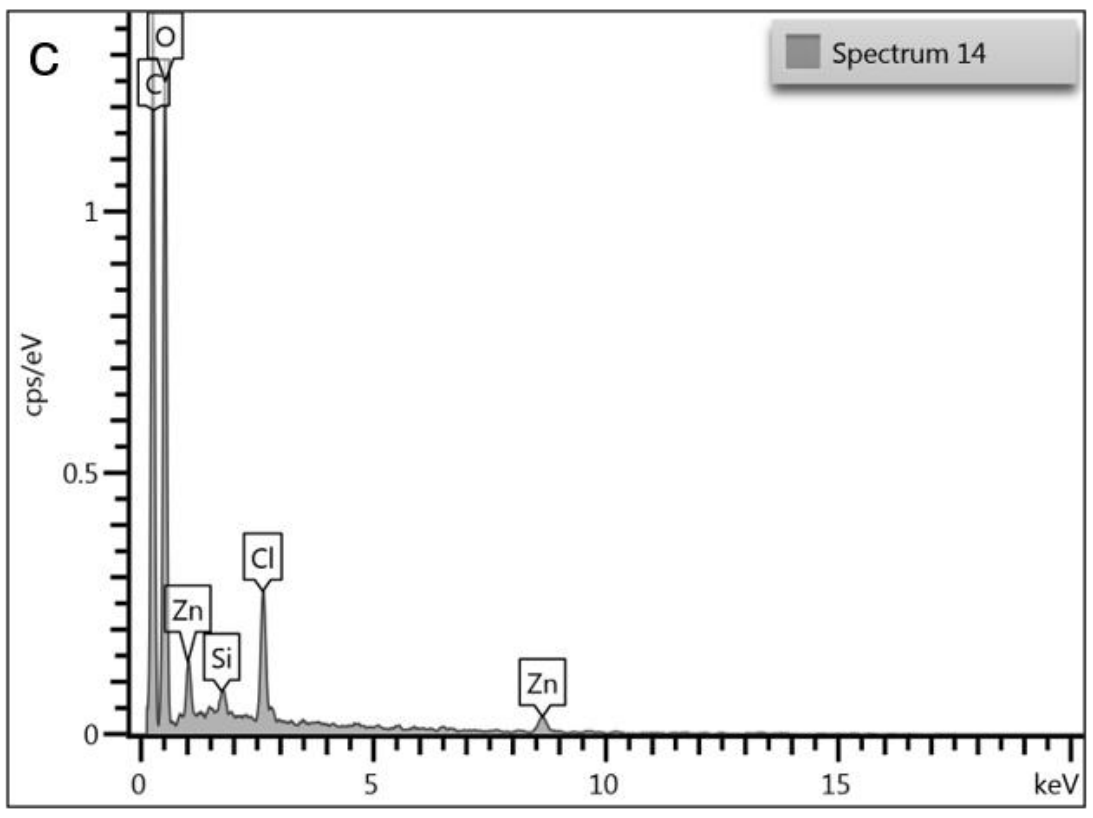

Spectrum Label	Spectrum 14	Spectrum 15	Spectrum 17
C	55.44	54.60	58.25
O	42.82	41.95	40.14
Si	0.11	0.13	0.12
Cl	1.04	2.13	0.82
Zn	0.59	1.18	0.66
Total	100.00	100.00	100.00

Figure S6. SEM micrographs at different magnifications of regenerated cellulose (a and b) and the corresponding EDX analysis (c) of ZnLA_CF obtained under mild treatment conditions.

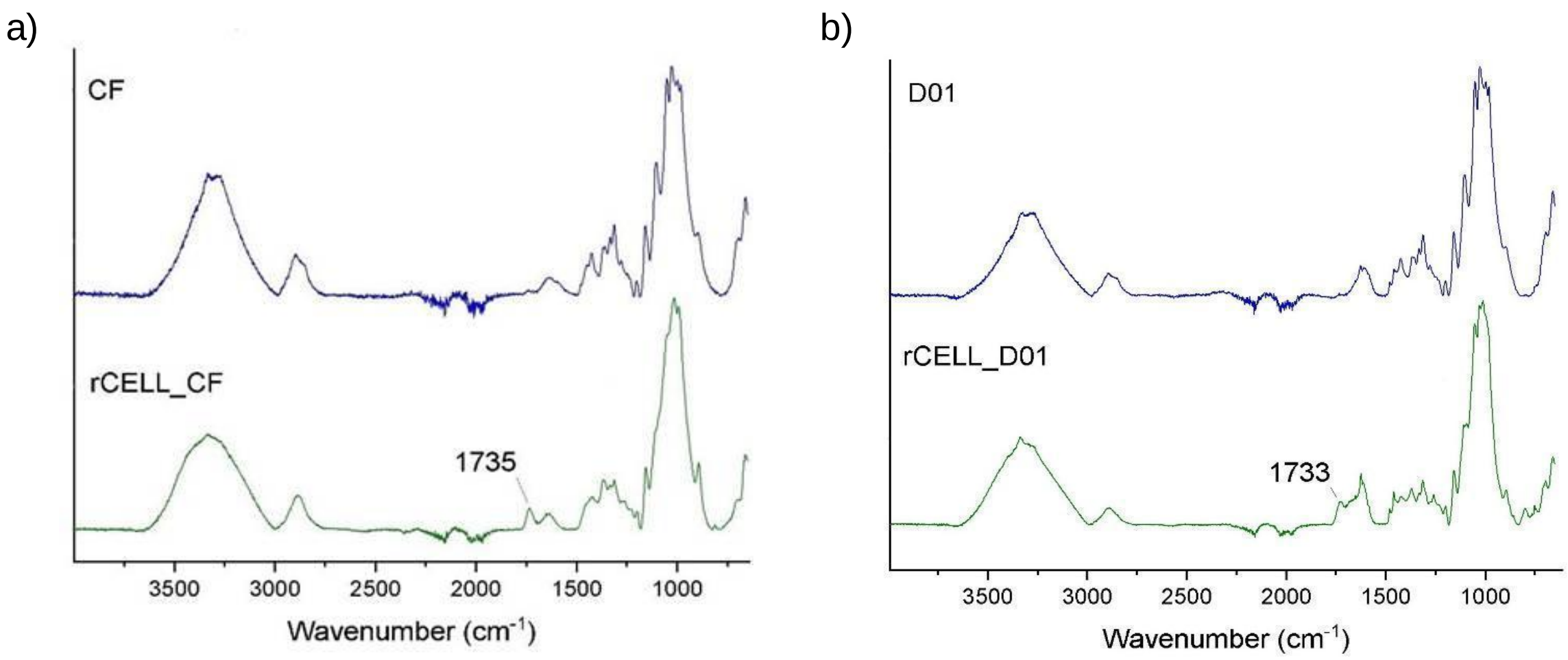

Figure S7: FTIR spectra comparing the pristine cellulosic materials with the corresponding recovered cellulose (rCELL): a) CF and b) D01.

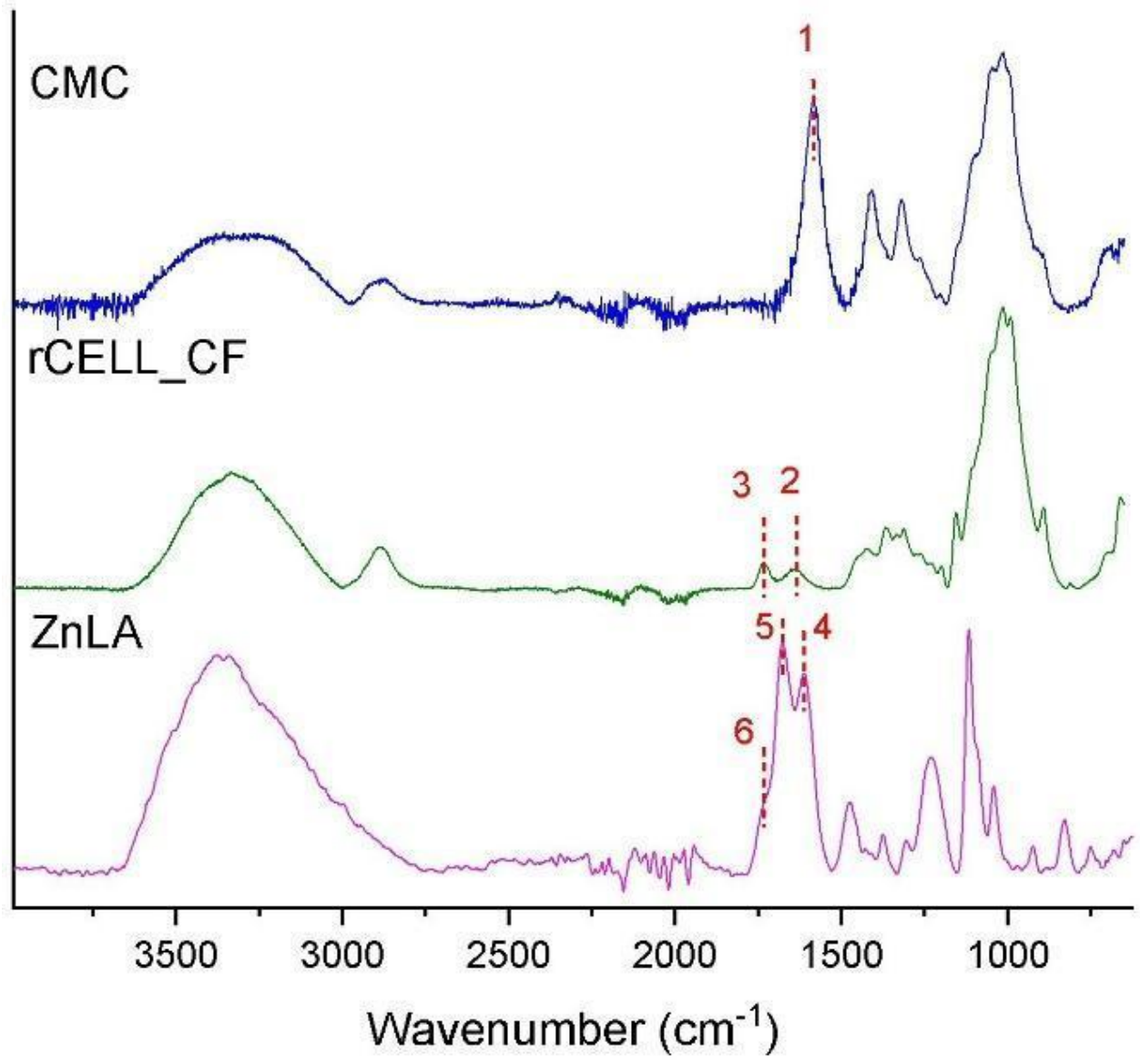

Figure S8: FTIR spectra of the cellulose recovered from the CF fraction, compared with carboxymethyl cellulose (CMC) and the ZnLA DES solvent as reference materials.

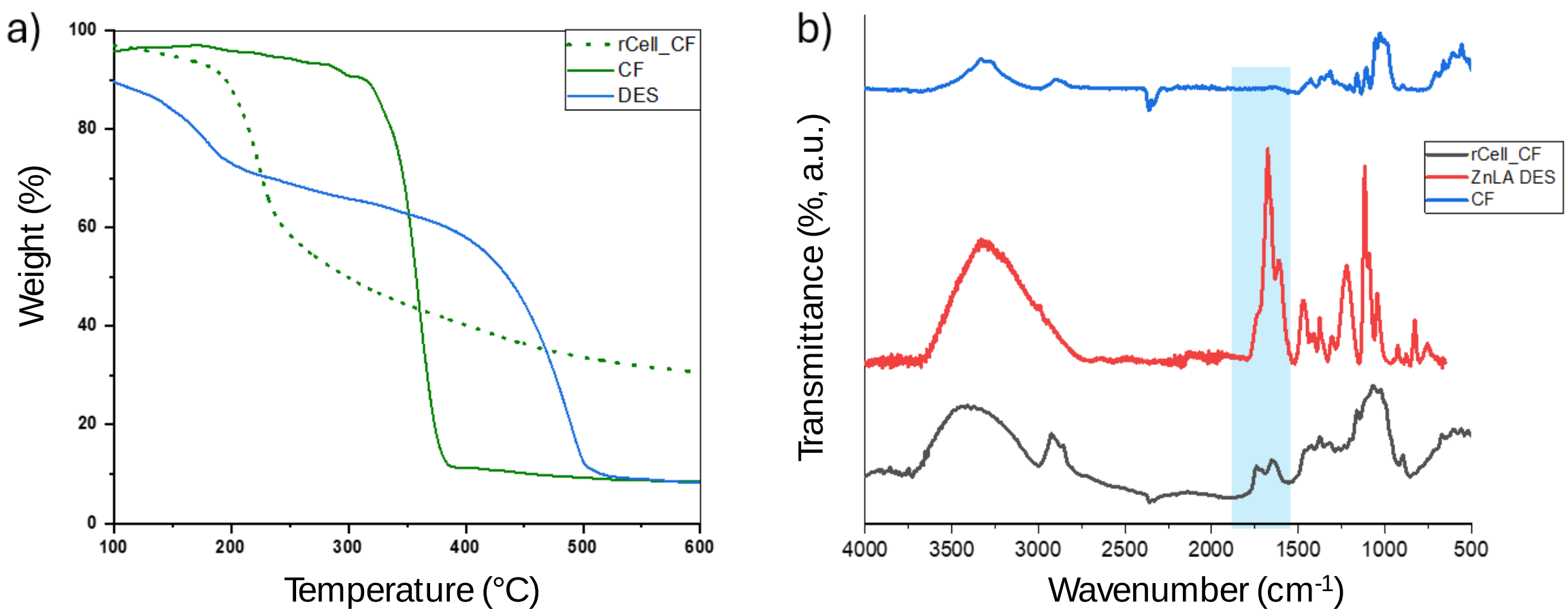

Figure S9. a) Thermogravimetric analysis (TGA) of CF, the cellulose recovered from CF, and the ZnLA deep eutectic solvent (DES); b) FTIR spectra of CF, the cellulose recovered from CF, and the ZnLA DES.

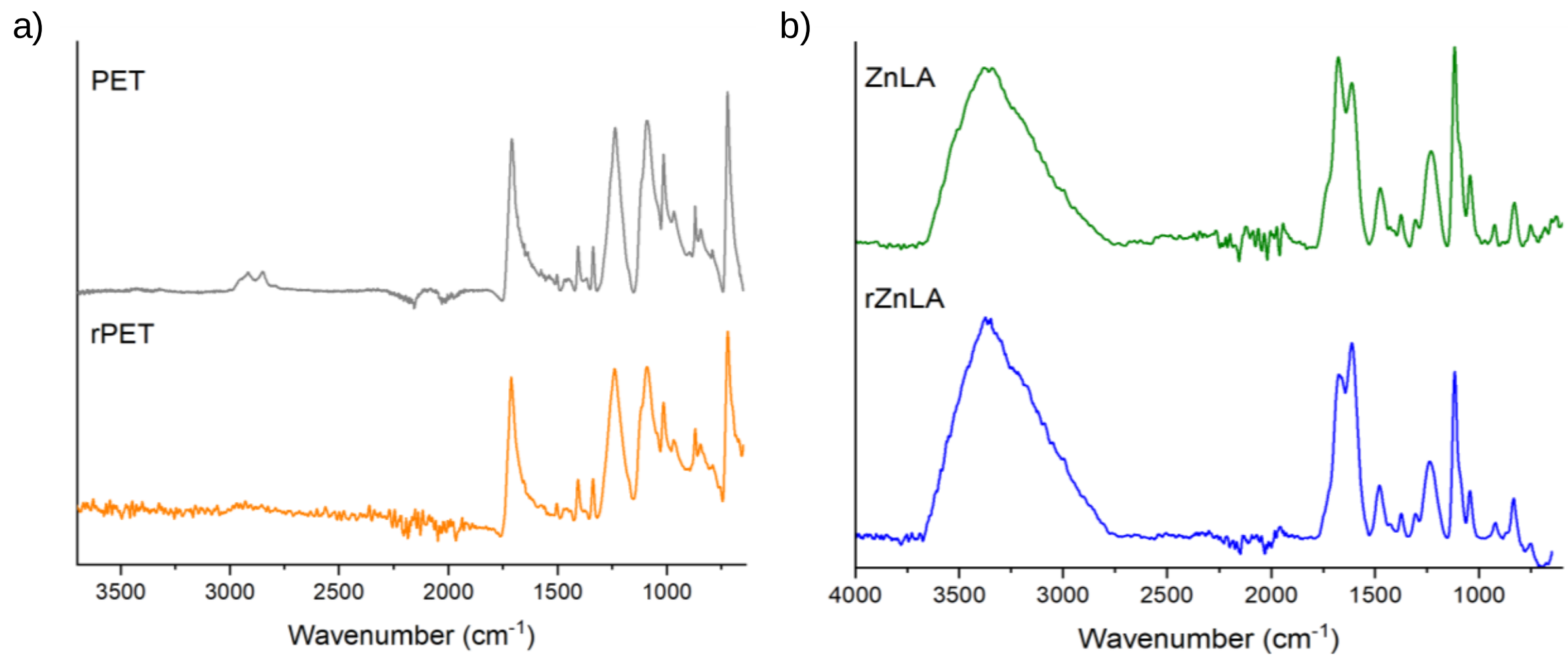

Figure S10: FTIR spectra of polyethylene terephthalate textile (a) and solvent (b) before and after treatment of synthetic fibers from D02 at 80 °C, 60 min, labelled PET and rPET, ZnLA and rZnLA, respectively.

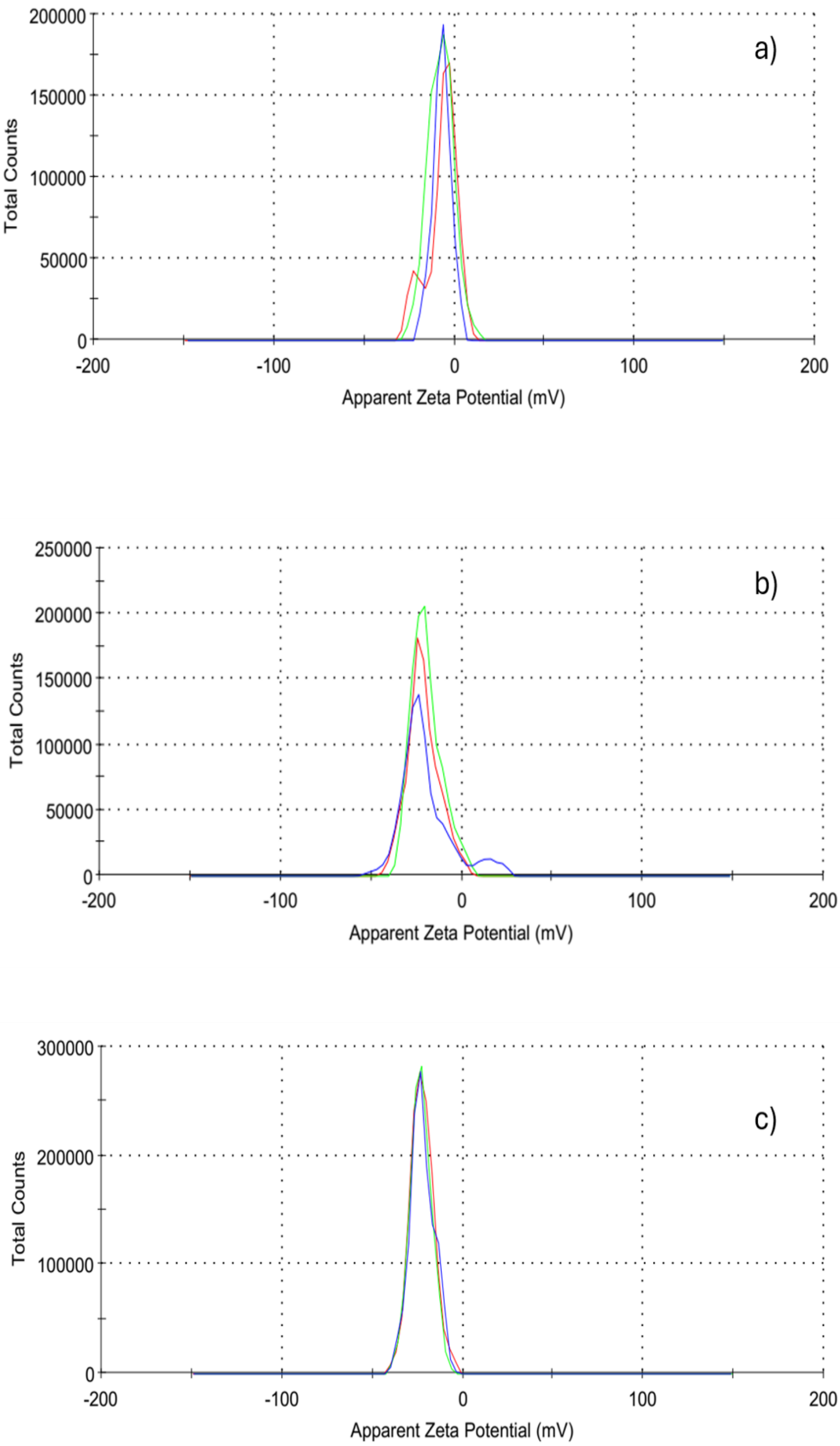

Figure S11. Zeta-potential analysis of samples: a) rCELL_CF (with ZnLA), b) CaLa_CF, c) ZnTA_CF, dispersed in water.

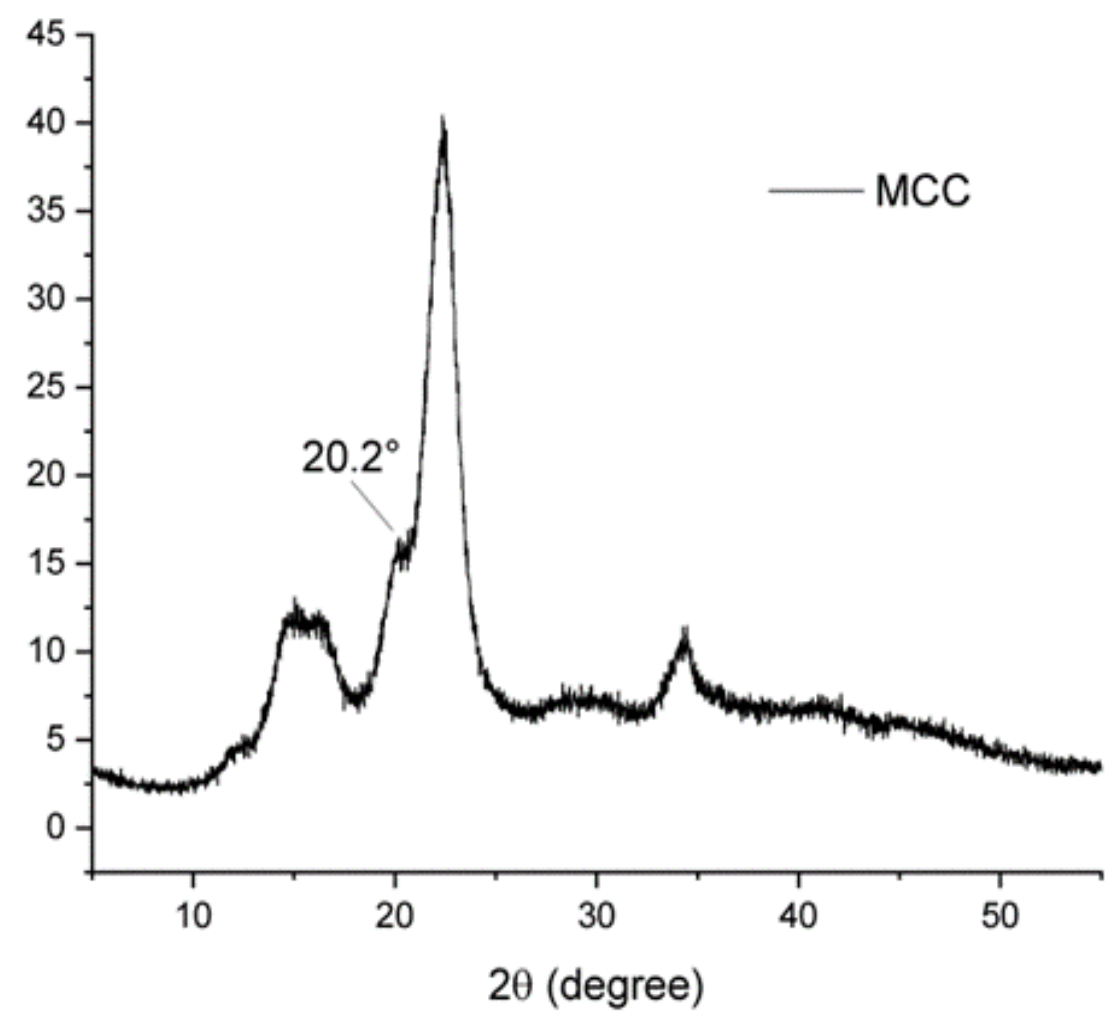

Figure S12: X-ray diffraction (XRD) pattern of microcrystalline cellulose (MCC) used as the crystalline cellulose reference.

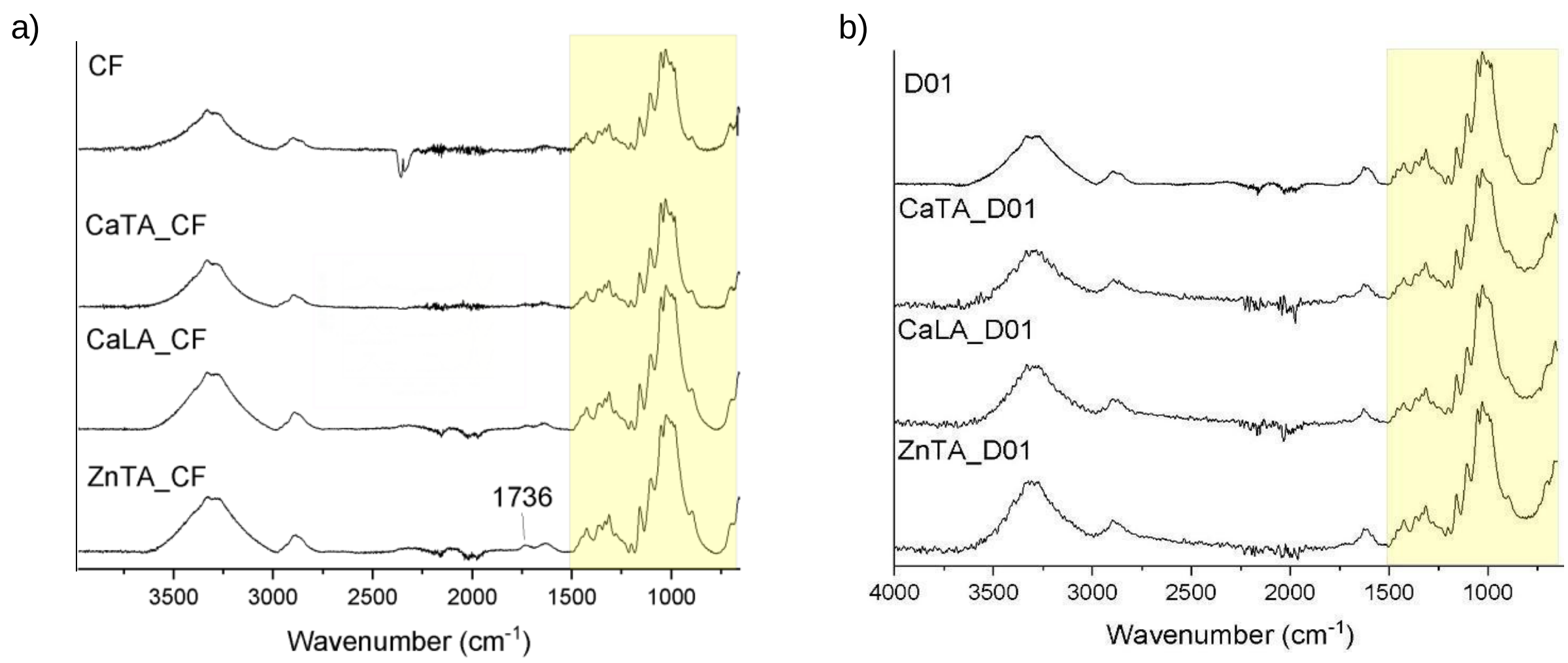

Figure S13: FTIR spectra of all cellulose nanocrystal (CNC) samples obtained by DES treatment, compared with the corresponding pristine materials: (a) CF and (b) D01. Yellow regions show retention of main spectral cellulose features.

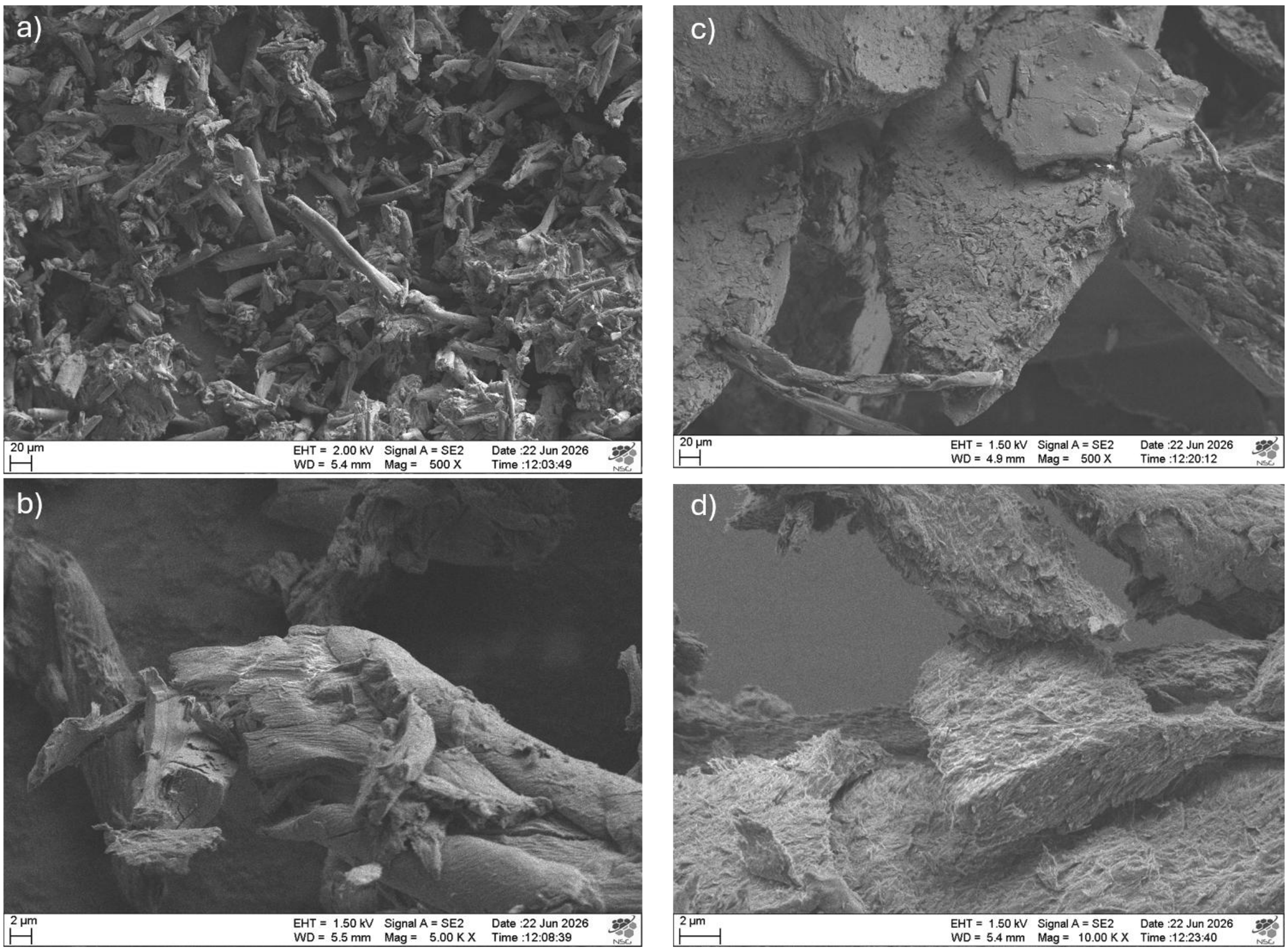

Figure S14. SEM micrographs of cellulose nanocrystals (CNCs) obtained from the CaLA_CF (a, b) and ZnTA_CF (c, d) systems at different magnifications (20 µm and 2 µm).

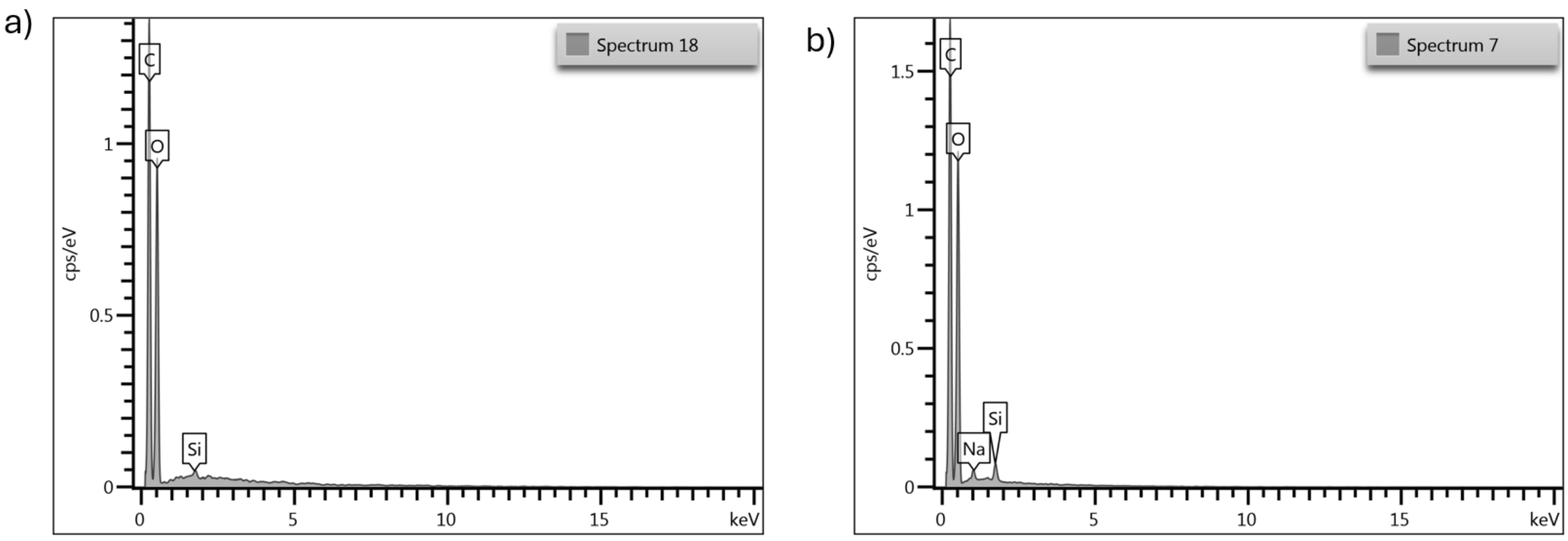

Spectrum Label	Spectrum 18	Spectrum 19	Spectrum 20
C	56.49	55.72	56.67
O	43.45	44.18	43.28
Si	0.06	0.10	0.05
Total	100.00	100.00	100.00

Spectrum Label	Spectrum 7	Spectrum 8	Spectrum 10
C	56.83	55.60	55.40
O	42.71	44.27	44.47
Na	0.22	0.10	0.07
Si	0.23	0.04	0.06
Total	100.00	100.00	100.00

Figure S15. EDX elemental analysis of cellulose nanocrystals (CNCs) obtained using the CaLA_CF (a) and ZnTA_CF (b) DES systems. For each sample, spectra were

recorded at three selected areas; the data from the two additional areas are reported in the corresponding tables.

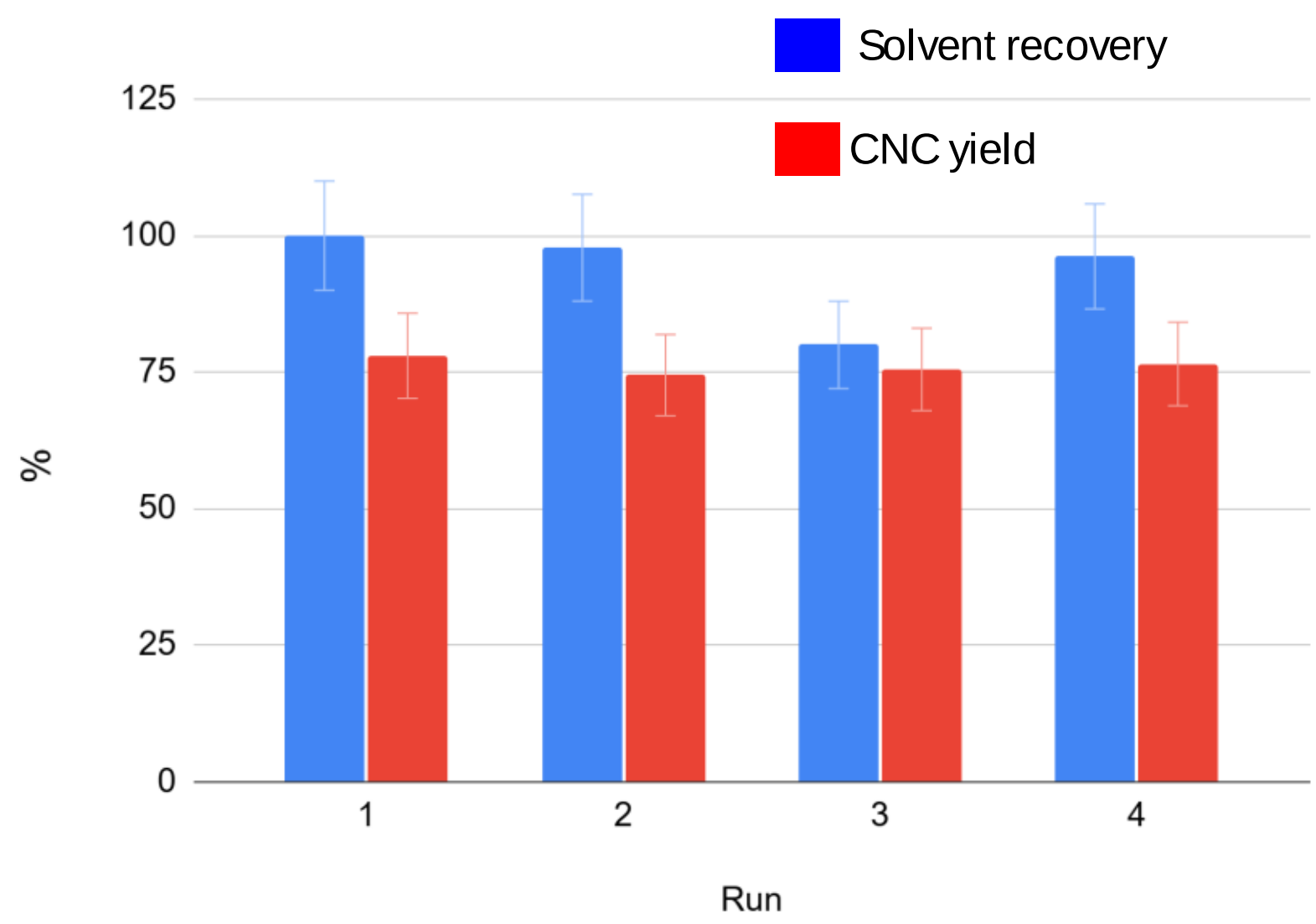

Figure S16. Solvent recovery and cellulose nanocrystal (CNC) yield over four consecutive cycles using the CaLA deep eutectic solvent (DES). Solvent recovery was determined gravimetrically after regeneration by rotary evaporation and is expressed as the percentage of the initial DES mass. CNC yield is reported relative to the initial cotton feedstock.

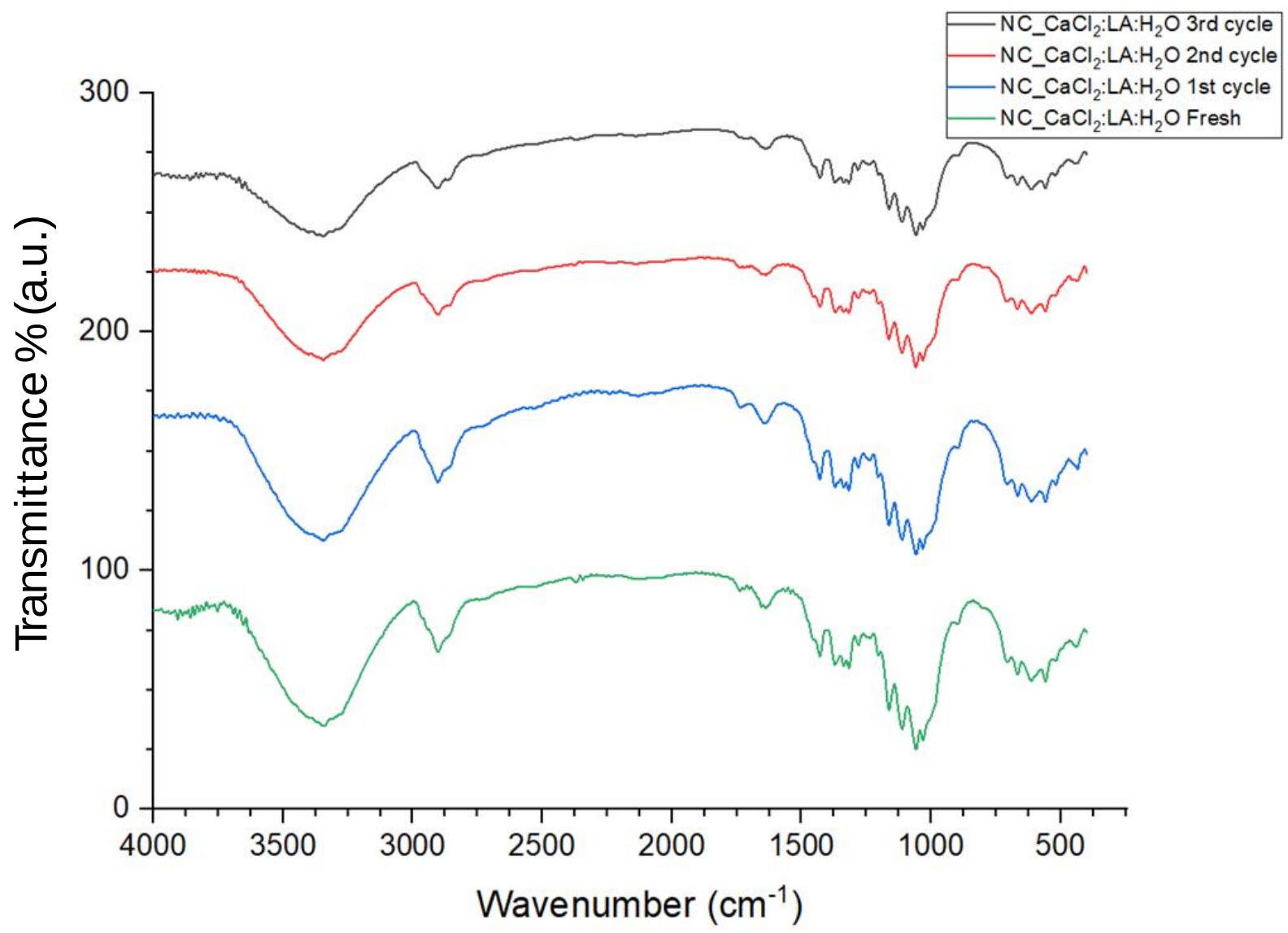

Figure S17. FTIR spectra of the CaLA deep eutectic solvent (DES) before use (fresh) and after three consecutive regeneration cycles. Following each nanocellulose (NC) extraction, the DES-containing liquid phase was regenerated by rotary evaporation under reduced pressure (45–50 °C, 100 rpm) to remove the excess water used as an

anti-solvent during NC precipitation. The recovered DES was then reconstituted to its original composition by water addition and reused in subsequent extraction cycles.

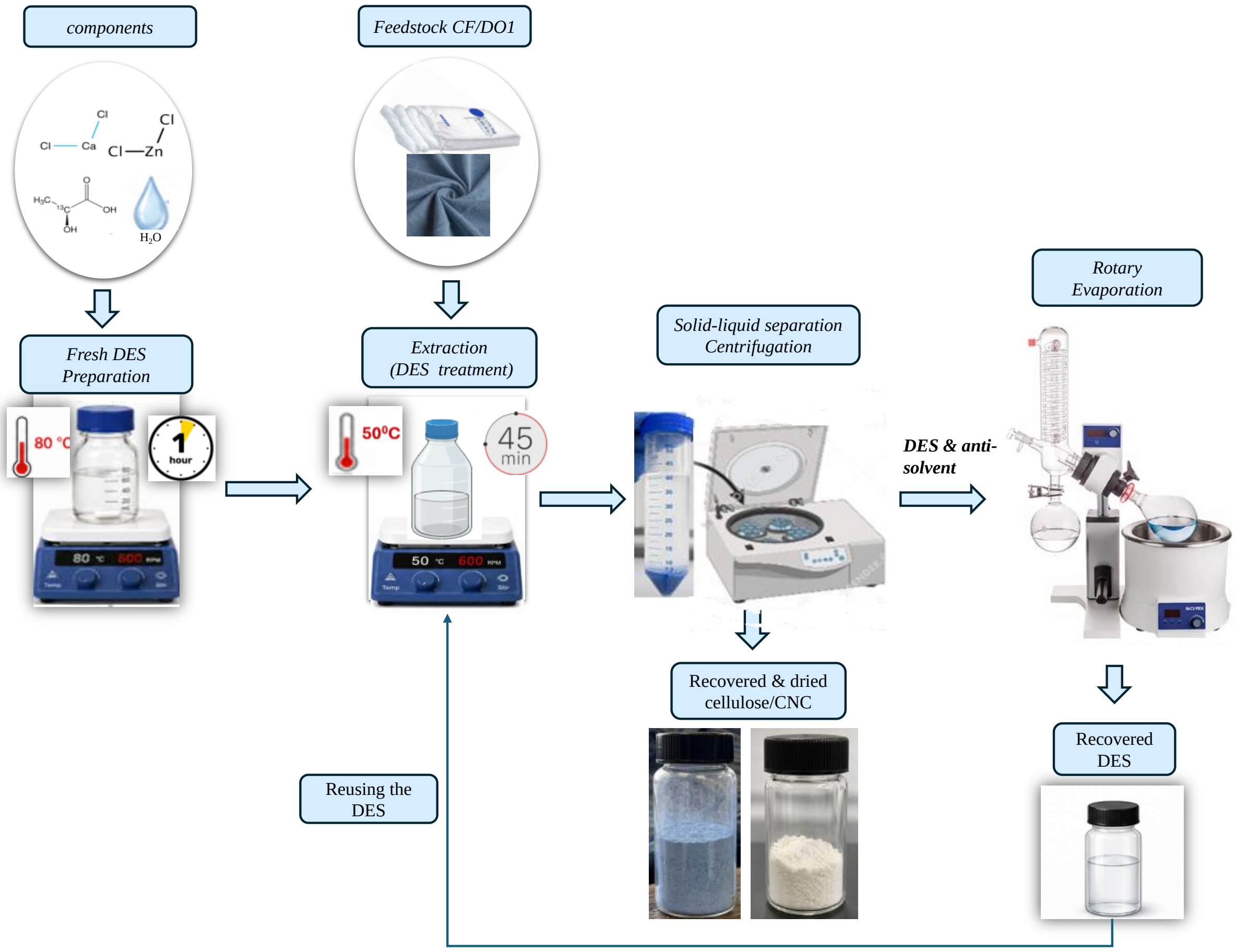

Scheme S1: Schematic illustration of the cotton recycling protocol using deep eutectic solvents (DESs). Fresh hydrated metal-based DESs are first prepared and employed for the extraction of cellulose from cotton feedstocks under mild conditions. Following precipitation with a non-solvent and solid–liquid separation by centrifugation, the recovered cellulose or cellulose nanocrystals (CNCs) are isolated, while the liquid phase containing the DES is subjected to rotary evaporation to remove water and ethanol. The recovered DES is then reconstituted to its original composition by the addition of water and reused in subsequent extraction cycles.

Table S1: Dissolution tests were performed on MCC using different DES compositions and molar ratios. The maximum cellulose loading that yielded a macroscopically homogeneous within 24 h at 80 °C was taken as the apparent dissolution limit.

**Name**	**Components**	**Molar Ratio**	**DES formation**	**MCC solubility (w/w DES%)**
CCCA	choline chloride:citric acid	1:3	x	
		1.5:3	x	
		2.5:3	x	
		2:2	x	
		3:2	x	
		4:2	x	
		5:2	x	
		6:2	√	x
CCCA_m1	choline chloride:citric acid:calcium chloride	1:6:2	x	
		2:6:2	x	
		3:6:2	x	
		4:6:2	x	
		5:6:2	x	
		6:6:2	x	
CCCA(M)	choline chloride:citric acid monohydrate	1:1	√	x
CCCA(M)_m1	choline chloride:citric acid monohydrate:water:calcium chloride	1:1:1:0	√	x
		1:1:1:0.2	x	
		1:1:2:0.2	x	
		1:1:6:0.6	x	
CCTA	choline chloride:tartaric acid	1:1	x	
		2:1	√	x
CCTA_m1	choline chloride:tartaric acid:water:calcium chloride	2:1:0:0.2	x	
		2:1:0.4:0.2	x	
		2:1:0.8:0.2	x	
		2:1:1.2:0.2	x	
CCLA	choline chloride:lactic acid	1:1	√	x
		1:2	√	x
		1:3	√	x
CaTA	tartaric acid:calcium chloride:water	1:1:6	√	x
CaLA	lactic acid:calcium chloride:water	1:1:6	√	x
ZnTA	tartaric acid:zinc chloride:water	1:1:6	√	x
-	-	-	-	-
ZnLA_01	zinc chloride:lactic acid:water	3:3:9	√	x
ZnLA_02	zinc chloride:lactic acid:water	4:3:9	√	1
ZnLA_03	zinc chloride:lactic acid:water	5:3:9	√	1
ZnLA_04	zinc chloride:lactic acid:water	6:3:9	√	1
ZnLA_05	zinc chloride:lactic acid:water	3:6:9	√	x
ZnLA_06	zinc chloride:lactic acid:water	4:6:9	√	x
ZnLA_07	zinc chloride:lactic acid:water	5:6:9	√	x
ZnLA_08	zinc chloride:lactic acid:water	6:6:9	√	1
ZnLA_09	zinc chloride:lactic acid:water	3:2:9	√	1
ZnLA_10	zinc chloride:lactic acid:water	4:2:9	√	4

ZnLA_11	zinc chloride:lactic acid:water	5:2:9	√	2
ZnLA_12	zinc chloride:lactic acid:water	6:2:9	√	x
ZnLA_13	zinc chloride:lactic acid:water	4:2:8	√	7
ZnLA_14	zinc chloride:lactic acid:water	4:2.5:8	√	10
ZnLA_15	zinc chloride:lactic acid:water	4:2.5:9	√	12
ZnLA_16	zinc chloride:lactic acid:water	1:3:8	√	9
ZnLA_17	zinc chloride:lactic acid:water	1:3:7	√	7
ZnLA_18	zinc chloride:lactic acid:water	2:1:9	x	x
ZnLA_19	zinc chloride:lactic acid:water	3:1:9	√	8
ZnLA_20	zinc chloride:lactic acid:water	4:1:9	√	1
ZnLA_21	zinc chloride:lactic acid:water	5:1:9	√	x
ZnLA_22	zinc chloride:lactic acid:water	6:1:9	√	x

Table S2. Comparative performance of reported cellulose-dissolving systems specifically applied to textile waste recycling. All processes listed were intended as lab-scale.

**Solvent System and recovery technique**	**Feedstock**	**Pre-treatment**	**Dissolution T (°C)**	**Dye effects**	**Load (feedstock/ solvent)**	**Cellulose Recovery (% yield)**	**Degree of Polymeri-sation (DP) variations (%)**	**Solvent recovery**	**Ref.**
ILs [Bmim]OAc/DMSO – wet spinning	Denim (blue, red)	10 wt% NaOH for decolorisation purposes	80	Color can be retained or removed; powdering required for efficient decoloration	Polymer concentration of 6 wt% in binary IL solvent system with B/D ratio of 20:80	>75	n/a	Feasible — Distillation	[1]
ILs<br>[Emim]Cl and [Emim]OAc with DMSO as cosolvent – wet spinning	Industrial cotton residue - brushing waste (1–10 mm)	None	100	No dye present; optical brightener from the residue was maintained in the filaments	5 wt% cotton waste in IL/DMSO solution (80/20, w/w)	n/a	Significant reduction	n/a	[2]
NMMO monohydrate – film casting	Denim waste	5h pre-swelling at 50 °C	110–120	Indigo dye degradation	10 wt% in NMMO solution (50% H_2O)	n/a	Mostly retained high DP values	Anti-oxidative agents required; Feasible	[3]
Ternary NMMO/DES [Cho]Cl/MA	Denim waste	None	80	DES alone could not dissolve denim due to dye; NMMO was required	2 wt% denim powder / ternary solvent	n/a	Mostly retained high DP values	>95% — distillation	[4]
DES<br>Choline chloride: Lactic Acid	Textile poly-cotton fabric (WP-CF), 65:35	None	110 (>2h)	Not addressed	n/a	>95%	Significant reduction	n/a	[5]
DES<br>ZnLA	Denim (blue)	None	50	Dye retained unchanged	10 wt% in ZnLA DES	n/a	n/a	Feasible	This work

References

[1] Y. Ma, B. Zeng, X. Wang, N. Byrne, *ACS Sustainable Chem. Eng.* **2019**, *7*, 11937–11943.
[2] B. B. de Brito, B. Krüger, L. S. da Silva, C. Marangoni, A. C. K. Bierhalz, *ACS Omega* **2025**, *10*, 19983–19991.
[3] M. A. Bagherjeri, A. N. M. A. Haque, H. Monhemi, M. Naebe, *Carbohydr. Polym.* **2024**, *346*, 122655.
[4] M. A. Bagherjeri, M. Bayattork, A. N. M. A. Haque, Z. Mahdavifar, F. Rezaie, Y. Zhang, M. Naebe, *J. Environ. Chem. Eng.* **2025**, *13*, 119944.
[5] I. Ara, A. Lopatina, M. Mänttäri, M. Kallioinen-Mänttäri, I. Anugwom, *Fibers Polym.* **2025**, *26*, 5759–5767
